# Endocytosis and non-canonical autophagy mediate extracellular histones cytotoxicity in vascular models of sepsis

**DOI:** 10.3389/fimmu.2025.1650789

**Published:** 2026-01-14

**Authors:** C. Garcés, P. Tascón, Luis Diago-Domingo, A. J. Ibáñez, G. Herrera, L. R. Rodríguez, K. Salewskij, G. Jonsson, J. M. Penninger, C. Romá-Mateo, F. V. Pallardó

**Affiliations:** 1 Departamento de Fisiología, Facultad de Medicina y Odontología, Universitat de València, València, Spain; 2 Centro de Investigación Biomédica en Red de Enfermedades Raras (CIBERER), Instituto de Salud Carlos III, Madrid, Spain; 3 Instituto de Investigación Sanitaria INCLIVA, València, Spain; 4 Unidad Central de Investigación de Medicina (UCIM), Universitat de València, València, Spain; 5 Stem Cells, Aging and Neurodegeneration Group, Department of Experimental Medical Science, Faculty of Medicine, Lund Stem Cell Center, Lund University, Lund, Sweden; 6 Institute of Molecular Biotechnology of the Austrian Academy of Sciences (IMBA), Vienna BioCenter (VBC), Vienna, Austria; 7 Vienna BioCenter PhD Program, Doctoral School of the University of Vienna and Medical University of Vienna, Vienna, Austria; 8 Department of Laboratory Medicine, Medical University of Vienna, Vienna, Austria; 9 Innovative Organoid Research, Helmholtz Centre for Infection Research, Braunschweig, Germany; 10 Department of Medical Genetics, Life Sciences Institute, University of British Columbia, Vancouver, BC, Canada

**Keywords:** sepsis, extracellular histones, endocytosis, non-canonical autophagy, HUVEC, blood vessel organoids, LC3B, LC3-associated endocytosis

## Abstract

**Introduction:**

Sepsis, a widespread global ailment, involves an exaggerated immune response, leading to hyperinflammation and immunosuppression. Extracellular histones, released during hyperinflammation as part of the defensive response against pathogens, significantly contribute to sepsis pathogenesis, compromising viability of the host’s endothelial cells and contributing to organ failure.

**Methods:**

This study explores the link between cytotoxic effects of extracellular histones and endocytosis mechanisms in human umbilical vein endothelial cells (HUVECs) and blood vessel organoids (BVOs) incubated with extracellular histones and different modulators of endocytosis mechanisms.

**Results:**

Exposure to various doses of purified extracellular histones in both HUVECs cultures and BVOs revealed sub-lethal doses leading to histone entry and colocalization with the autophagy mediator LC3B, whereas high doses induced cytotoxicity. Incubating cells or organoids at low temperature before histone exposure prevented entry, reducing colocalization with LC3B and cell death; moreover, inhibition of clathrin-mediated endocytosis abrogated histone entry into HUVECs and prevented their cytotoxic effects, whereas inhibition of caveolin-mediated mechanisms had no effect.

**Discussion:**

In summary, this study offers insights into histones’ cytotoxicity and functional interactions with the LC3B-mediated, non-canonical autophagy pathway, enhancing our understanding of the molecular bases of sepsis pathophysiology within HUVEC and blood vessel organoids.

## 1 Introduction

Sepsis is characterized by a systemic exacerbated inflammatory response against infection, due to the dysregulation of intrinsic mechanisms. During infection, the immune system identifies a series of molecular patterns only present in the pathogen, known as PAMPs (Pathogen-associated molecular patterns). Besides microbial molecules, the immune system also detects DAMPs (Damage-Associated Molecular Patterns) which are produced by cell death processes in the infected host. DAMPs and PAMPs are recognized by PRRs (Pattern-Recognition Receptors) (1). Contact between DAMPs, PAMPs and these specific receptors leads to the activation of diverse signaling pathways which end in the activation of genes related to inflammation, immune response, and cellular metabolism (2).

Histones are globular proteins with a strong basic character and are very conserved in all vertebrates. In humans, the basic structural unit of chromatin is formed by a core of four different histones (H2A, H2B, H3, and H4) in a dimeric way, forming an octamer bound to a strand of DNA of approximately 147 base pairs which wraps this octameric core, leading to the basic structural unit of chromatin, the nucleosome. However, when an organism faces stress such as tissue damage or injury that causes cell death, histones and other nuclear proteins can be released into the extracellular space (3). Once in the extracellular space and in the bloodstream, histones can act as DAMPs, activating the immune system and causing cytotoxicity in the body’s cells, which can lead to necrosis and increased release of more DAMPs in a positive feedback process. This release of histones and other nuclear proteins like HMGB1, which has been associated with the pathogenesis of different diseases such as trauma, autoimmune diseases, or sepsis (4), has been observed in patients with different pathologies. In this context, systemic immune responses are activated, leading to the release of proinflammatory cytokines and chemokines (5). During progression of sepsis, the release of extracellular histones is exacerbated due to plasma membrane rupture leading to inflammation, tissue damage, thrombosis and organ failure, among others (6, 7). There are two mechanisms through which it is postulated that histones exert their extracellular actions. The first one depends on Toll-like receptors (TLRs) (8). TLR-2 and TLR-4, which are believed to interact with histones, are postulated to promote the activation and recruitment of neutrophils, monocytes, and macrophages, as well as to trigger the release of proinflammatory cytokines related to sepsis (3). The other mechanism of cytotoxicity of histones is directly driven by the positive charges of arginine and lysine residues present in histone tails, which allows them to preferentially bind to the polar heads of membrane phospholipids through electrostatic interactions, inducing plasma membrane permeabilization and disrupting calcium signaling pathways (9). In fact, histones that underwent post-translational modifications to become citrullinated exhibited reduced cytotoxicity towards endothelial cells compared to non-citrullinated histones, did not impact cell viability, and did not induce oxidative stress due to their reduced positive charges (10). Extracellular histones have also been found in the plasma of septic patients and their impact has also been studied (7), corroborating the need to further dissect the molecular mechanisms in which they are involved during sepsis progression but also making promising biomarkers for the identification and diagnosis of sepsis and septic shock (11–13).

There are several types of cellular models for studying sepsis, including cell culture models like HUVEC and organoid-like models (14). Organoids involve the creation of miniature organ systems that can mimic the function and gross morphology, through cellular architecture, of multiple cell types in a specific organ (15). However, conventional, two-dimensional cell cultures do not reflect the complexity of vascular endothelium as they are a monolayer of a unique cell type, therefore, the development of self-organizing 3D human blood vessel organoids (BVOs) from human pluripotent stem cells (hPSCs) is considered one of the most significant advancements in the field of modeling human blood vessels. These BVOs accurately mimic the functional, morphological, and molecular characteristics of human microvasculature, complete with an endothelium, a continuous basement membrane, and surrounding mural cells (16, 17). The utilization of the BVO system thus provides a more sophisticated approach to interrogate disease pathogenesis, serving as a human-based and highly scalable drug-screening platform.

All this evidence highlights the fundamental role that histones play in the development of sepsis as one of the most relevant mediators, modulating the intense pro-inflammatory and procoagulant responses characteristic of sepsis (18, 19) and their potential as biomarkers (11–13). Likewise, the different mechanisms through which histones cause cytotoxicity in cells continue to be explored to gain a better understanding of the biological responses that are affected by the action of histones. Among them, as we have already shown, the autophagy pathway must be highlighted (20). Here we demonstrate that extracellular histones are cytotoxic in two different experimental vascular models and that their effects are dependent on their entry into cells, where they interact with the autophagy mediator LC3B. Thus, our results point to a potential role of LC3-associated endocytosis (LANDO), a non-canonical pathway of autophagy, as one of the internalization mechanisms relevant for histone-mediated cytotoxicity in endothelial cells.

## 2 Results

### 2.1 Extracellular histones are cytotoxic for HUVEC and BVOs at high doses

Given that it has been demonstrated that the addition of extracellular histones affects cell survival in a dose-dependent manner (20), we aimed to investigate whether extracellular core histones (a mixture of histone H2A, H2B, H3 and H4) and fluorescent histone H1 (so on called H1-488) had detrimental effects on two cellular models: HUVEC and human induced pluripotent stem cell derived blood vessel organoids (BVOs). A mixture of purified core histone proteins, and H1–488 were used at concentrations ranging from 10 to 500 µg/mL and added to both models for either 4 hours (in the case of core histones) or 24 hours (in the case of H1-488). Cell survival was analyzed using an apoptosis study kit for flow cytometry. To validate that the observed cytotoxic effects were histone-specific, HUVEC were also exposed to high concentrations of BSA (see **Supplementary Figure S1**).

**Figure 1:**
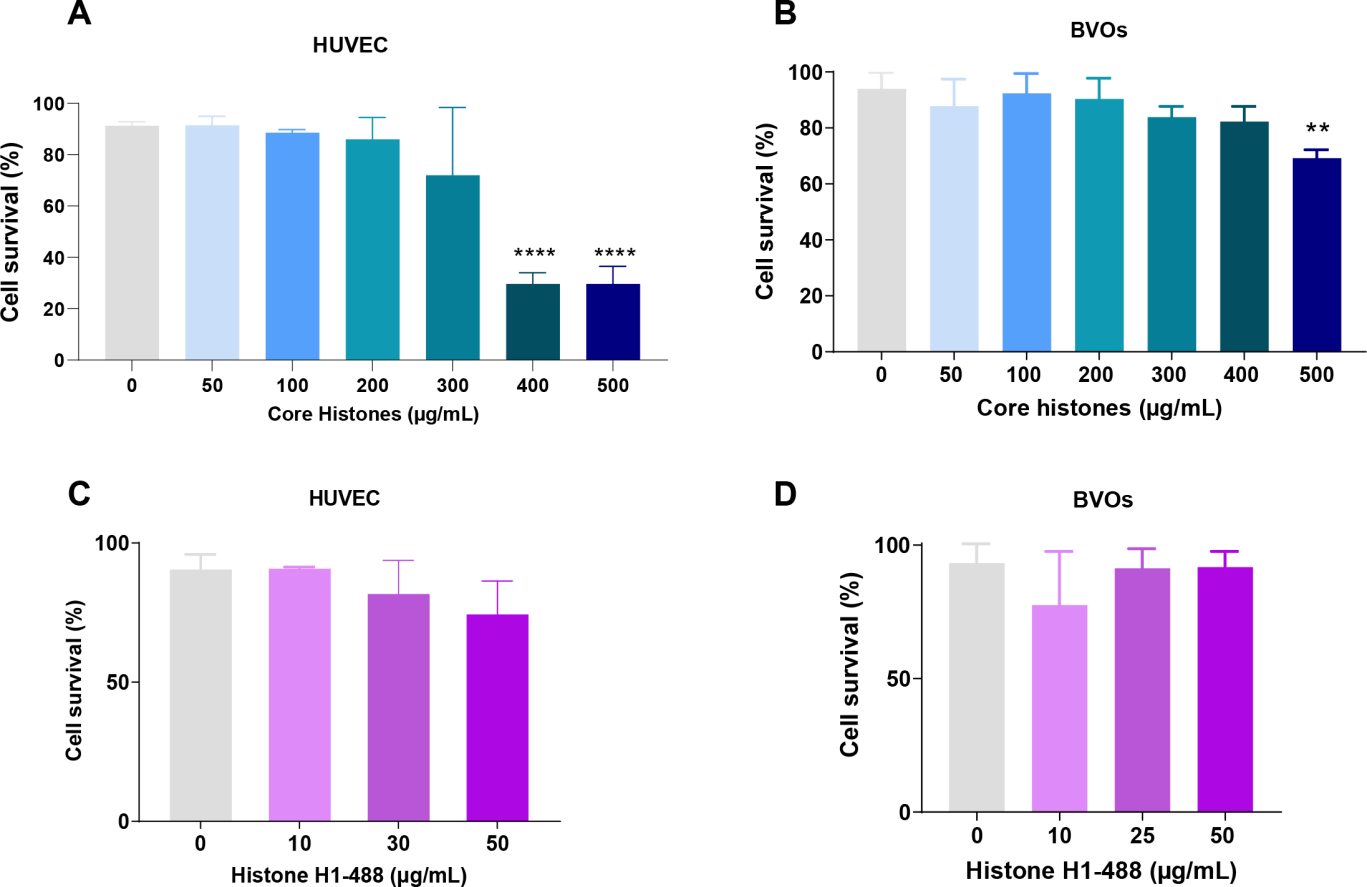
Analysis of histone-mediated cytotoxicity on HUVEC and blood vessel organoids (BVOs). Both systems were incubated in the absence (control) or presence of several concentrations of core histones **(A, B)** for 4 hours or histone H1-Alexa488 **(C, D)** for 24 hours. After the indicated incubation times, cells were harvested, labelled using an Annexin V Kit and subsequently analysed by flow cytometry. The mean values are shown as bars, expressed as a percentage ± the standard deviation (n=3). Statistical significance was considered at a p-value < 0.05, calculated in all cases using a one-way ANOVA statistical test indicated by “**” when p<0.01, “***” when p< 0.001 and “****” when p<0.0001.

**Figure 1** demonstrates dose-dependent cytotoxicity of histones dependent on the subtype and cellular model. For core histones, concentrations higher than 400 µg/mL achieved a decrease in cell survival. However, no significant changes were observed in terms of cytotoxicity induced by histone H1-A88 treatment in HUVEC. Despite not being statistically significant, a trend towards a reduced cell viability in HUVEC can be observed from concentrations of 50 µg/mL onwards. To gain a more insightful perspective on the biological consequences of the interaction between circulating nucleoproteins and the endothelium, resembling the complex pathophysiological environment in sepsis, and to validate the previously obtained results on mono-cell cultures, we employed a human blood vessel organoid model, a more complex, pluricellular system, as previously described in (16, 17). For this purpose, at least 16 organoids per condition were subjected to treatments with core histones for 4 hours or with histone H1–488 for 24 hours.

**Figure 1B** indicates that core histones can cause a significant reduction in survival at high concentrations, starting from 500 µg/mL. However, histone H1–488 by itself was unable to reduce viability (21, 22). Despite showing an overall higher resistance to histone-mediated toxicity, the organoids confirm the trend observed in HUVEC as well as previous results obtained by our group and others (16, 17).

Therefore, we were able to confirm the cytotoxic potential of core histones in a dose-dependent fashion. These findings underscore the harmful impact of extracellular histones on endothelial cells, particularly in the context of sepsis.

### 2.2 Extracellular histones enter HUVEC cells via clathrin-mediated endocytosis

Previous studies have investigated the ability of histones to penetrate cells (21, 22). However, few have elucidated the underlying mechanisms. Recent studies by Wang et al. (23) have provided insights into the internalization of fluorescently labelled nucleosomes in HeLa cells. They demonstrated that nucleosomes initially interact with the cell membrane through nonspecific, non-electrostatic interactions with the positively charged histone tails, and subsequently enter the cells through clathrin- or caveolin-dependent endocytosis (24). Therefore, we aimed to reveal the mechanism of histone internalization in our models.

**Figure 2:**
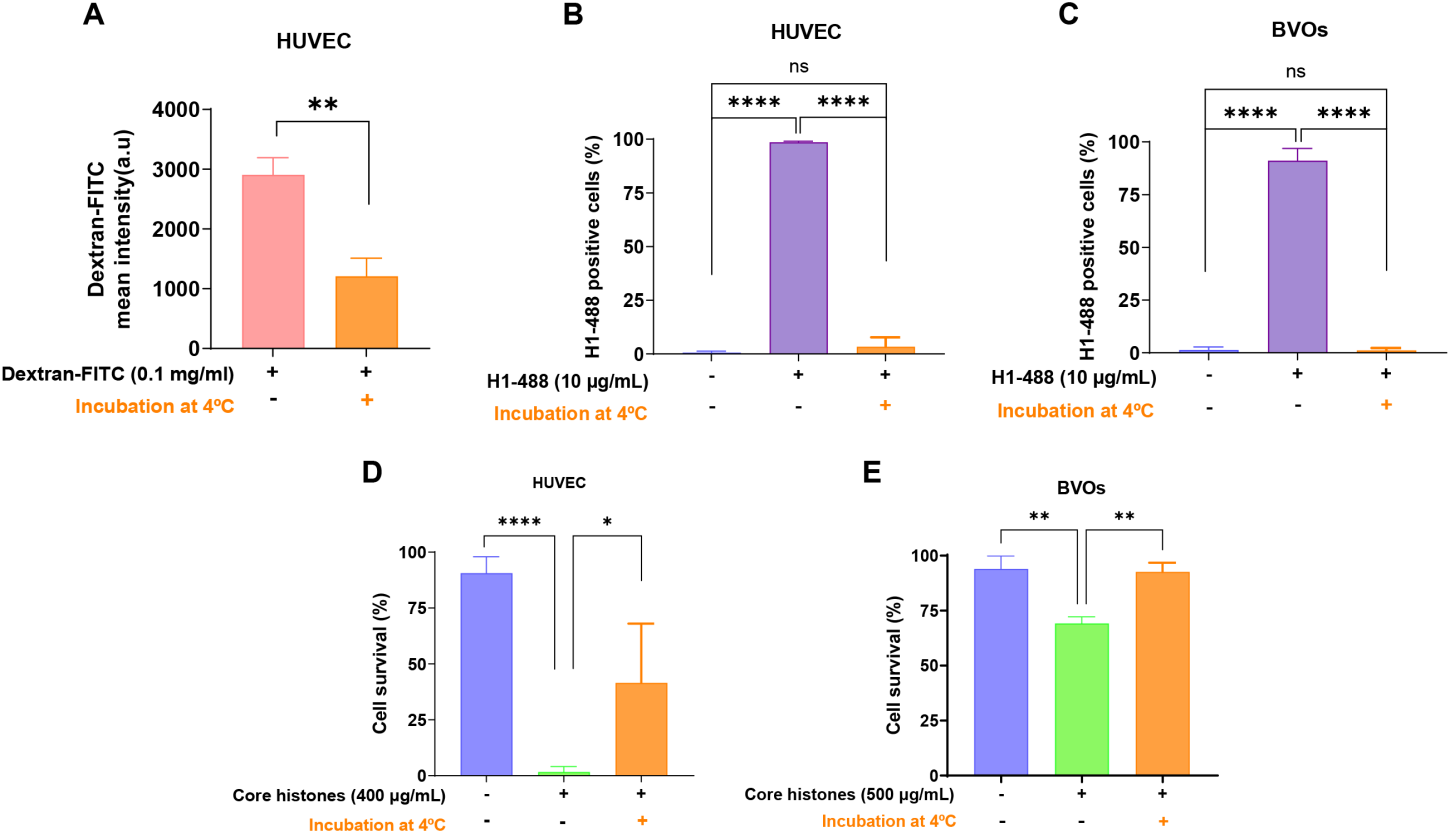
Blocking endocytosis prevents histone entry to HUVEC and Blood Vessel Organoids (BVOs) and reduces histone-induced cytotoxicity. **(A)** 1 hour serum-starved HUVEC were incubated with 0.1 mg/ml of 40.000 mw Dextran-FITC at 37°C or 4°C for 2 hours. Bars represent Dextran-FITC mean intensity measured by flow cytometry. HUVEC **(B)** or BVOs **(C)** were incubated with 10 μg/mL of H1-488 for at 37°C 24h (control condition), as well as blocking endocytosis by incubating cells on ice. Graphs show relative percentage of Alexa488-positive live cells as determined by flow cytometry. B) Serum starved (1h) HUVEC **(D)** or BVOs **(E)** were incubated in the presence of 400 or 500 μg/mL of core histones, respectively, for 4h at 37 °C in the case of control condition and on ice in the condition of blocking endocytosis. Graphs show relative cell survival rates as determined by Annexin V staining and subsequent flow cytometry. Mean survival values are presented as bars, expressed as a percentage ± standard deviation (n=3), with statistically significant differences considered when p<0.05, calculated using T-test or Anova one way, “*” from p<0.05, “**” from p< 0.01 and “***” when p<0.001.

In a first attempt to elucidate the consequences of exposure to extracellular histones, we verified that the histones added to cell- and organoid cultures were indeed being incorporated into the cells (**Figure 2**). For this, HUVEC and BVOs were treated with histone H1-488 (10 µg/mL) for 24 hours at 37°C (standard treatment conditions). In parallel, cells and BVOs were treated with 10 µg/mL of H1–488 and incubated at 4°C, a method previously used by Wang and collaborators to abrogate endocytosis entry of elements into cells (23). Ice cold incubation is a convenient and established method for inhibiting endocytosis in an unspecific way (25) and it has proved to yield good evidence for endocytosis studies (26, 27) and even similar results to using specific chemical inhibitors (28). Likewise, we validated the effect of incubating HUVEC at 4°C by analyzing the uptake of dextran 40.000 mw (a typical cargo of micropinocytosis) (**Figure 2A**). Subsequently, cells were collected and the FITC signal from dextran or histone treatment was analyzed using flow cytometry.

As shown in **Figures 2B, C**, incubation with the fluorescently-tagged histone leads to a successful internalization, evident by the high percentage of FITC-positive cells. This indicates that histone H1–488 is indeed entering the cells and organoids. Pre-incubation at 4°C on the other hand leads to a drastic reduction in FITC-positive cells, comparable to the histone-untreated condition. These results indicate that histones enter cells mainly through an endocytosis-dependent mechanism.

Next, we wanted to determine if blocking histone entry had a beneficial effect on cell and organoid survival, confirming that the cytotoxic effects observed with core histone treatments depend on histone entry through endocytosis mechanisms. To unravel this, HUVEC and organoids were treated with 400 µg/mL or 500 µg/mL of histone core for 4 hours. In the control condition, this incubation was carried out at 37°C, while in the endocytosis-blocking condition, the cells were incubated at 4°C.

**Figure 3:**
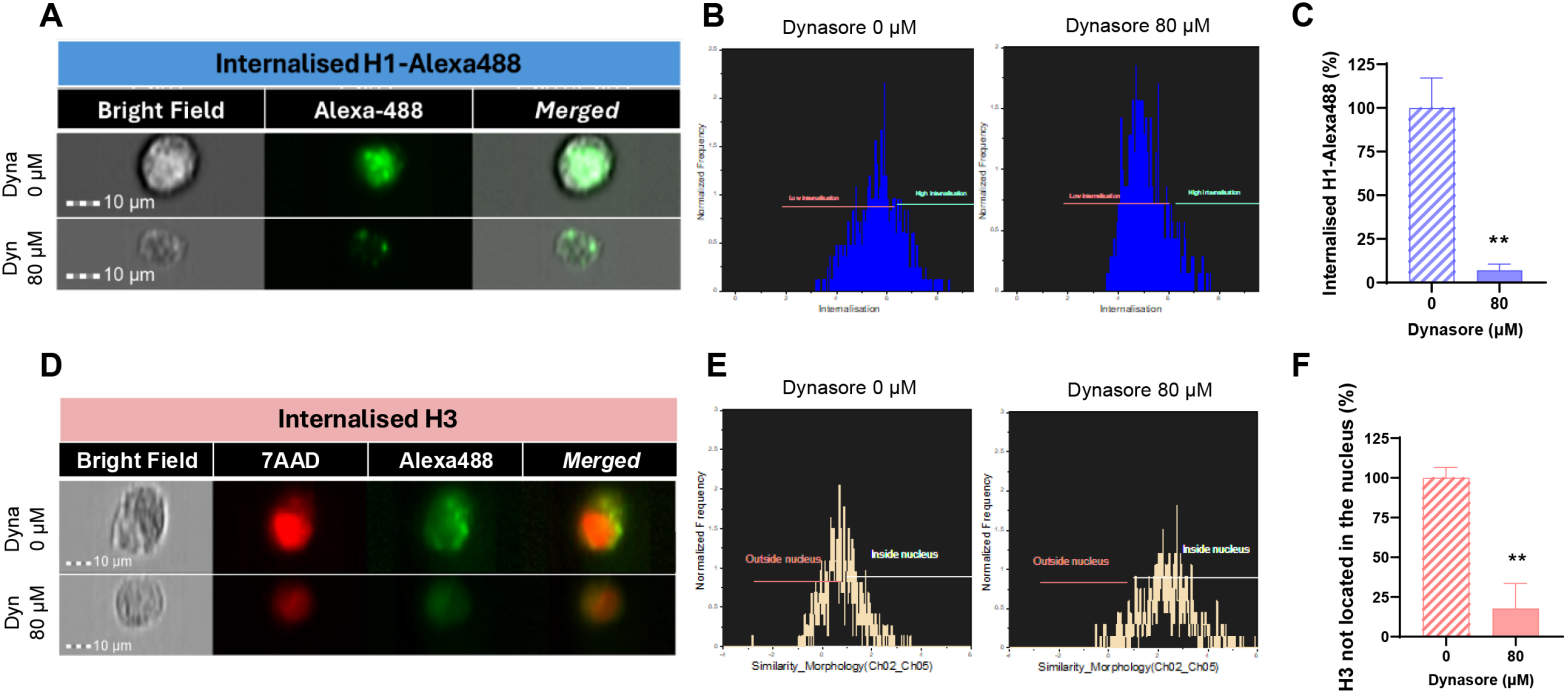
Effect of blocking clathrin-mediated endocytosis with dynasore on the internalization of extracellular histones in HUVEC. Representative images of Alexa488-conjugated H1 **(A)** or H3 **(D)** internalization in HUVEC cells incubated with dynasore (0 and 80 μM) and 10 μg/ml H1-Alexa488 **(A)** or 50 μg/ml Core Histones (H2AB, H3 and H4) **(D)** obtained with the INSPIRE software (ImagesStreamX Mark; Amnis-Merck Millipore). Red fluorescence corresponds to cellular nuclei stained with 7AAD and green fluorescence corresponds to histone H1-Alexa488 **(A)** or histone H3 stained with an Alexa488-conjugated primary antibody. Magnification 40X. 10 μm scale bars **(B)** Histograms generated using the internalization wizard of the IDEAS software and **(E)** histograms resulting from the application of the internalization algorithm and the similarity feature to differentiate the green marker located in the nucleus from that not located in the nucleus. **(C, F)** Quantification of H1-Alexa488 internalization **(C)** or H3 located in the cytoplasm **(F)** of HUVEC using the internalization wizard provided by IDEAS software. Bars represent mean values as a percentage ± the standard deviation (n=3), considering significant differences compared to the control (dynasore 0 μM) when p<0.05 (T-test); “**” when p<0.01.

**Figure 4:**
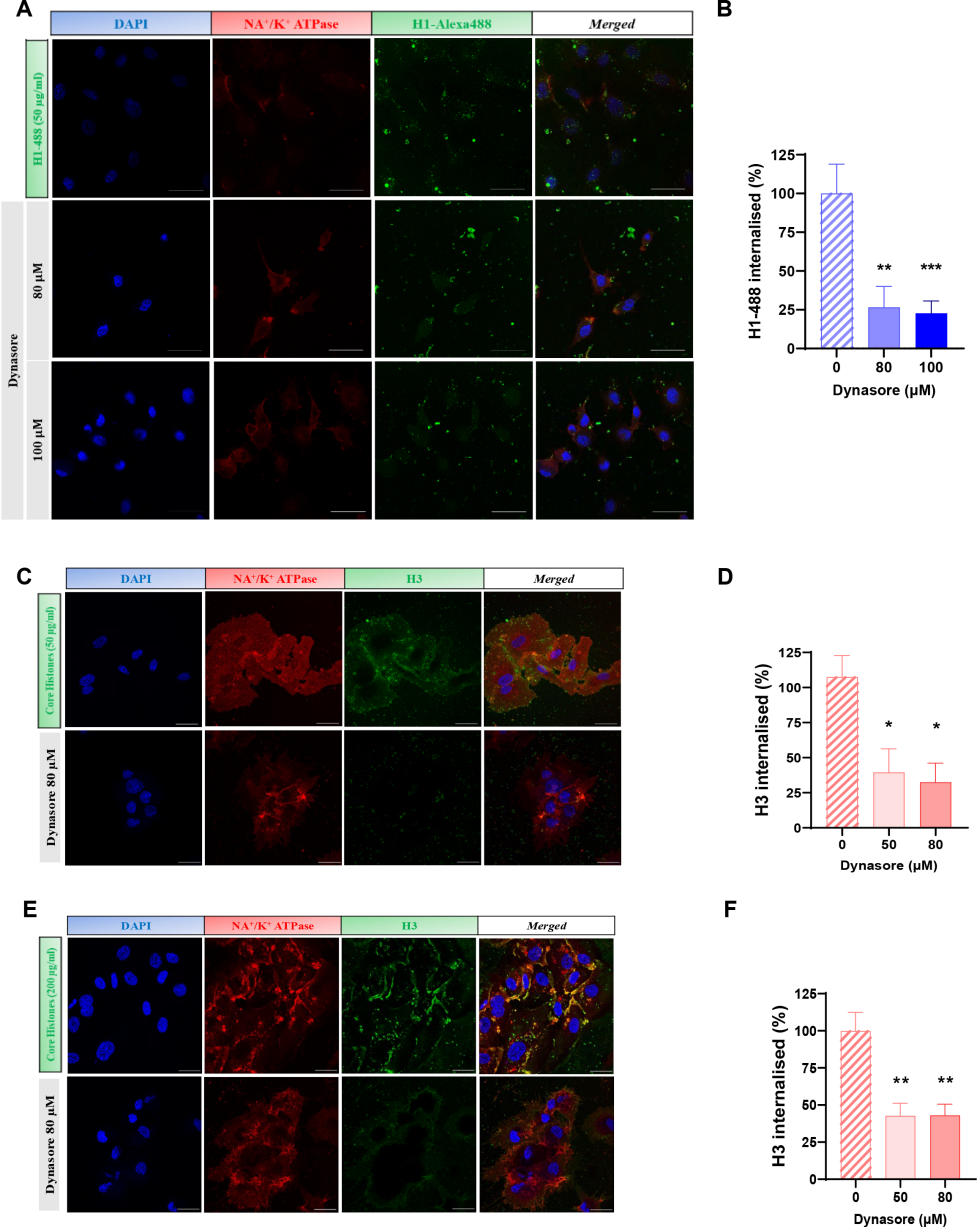
Confocal microscopy analysis of clathrin-mediated endocytosis inhibition with dynasore. **(A, C, E)** Representative images of HUVEC treated with with dynasore (0, 50, 80 and 100 μM) and 50 μg/ml histone H1-488 **(A)** or 50. μg/ml **(C)** or 200 μg/ml **(E)** Core Histones (H2AB H3 and H4). Blue fluorescence corresponds to cell nuclei by DAPI staining, red to Na+/K+ ATPase by binding to a secondary antibody conjugated to Texas Red fluorophore and the green one with the fluorescent histone H1-488 **(A)** or histone H3 by staining with a primary antibody conjugated to the fluorophore Alexa-48 (**C, E**). In addition, a fourth column is shown with the fusion of the three channels used. Magnification 63X. 40μm scale bars **(D)** Quantification of the internalisation of histone H1-488 **(B)** or H3 (**D, F**) in HUVEC using the Cell Profiler software. The bars show the mean values ± the standard deviation of the percentage of internalisation using CellProfiler 4.2.8 release (20 images per condition from 3 independent experiments). Statistically significant differences were considered with respect to the control (Dynasore 0 μM) when p<0.05, calculated using One-way Anova indicated by “*” when p<0.05 and “**” when p<0.01.

**Figure 5:**
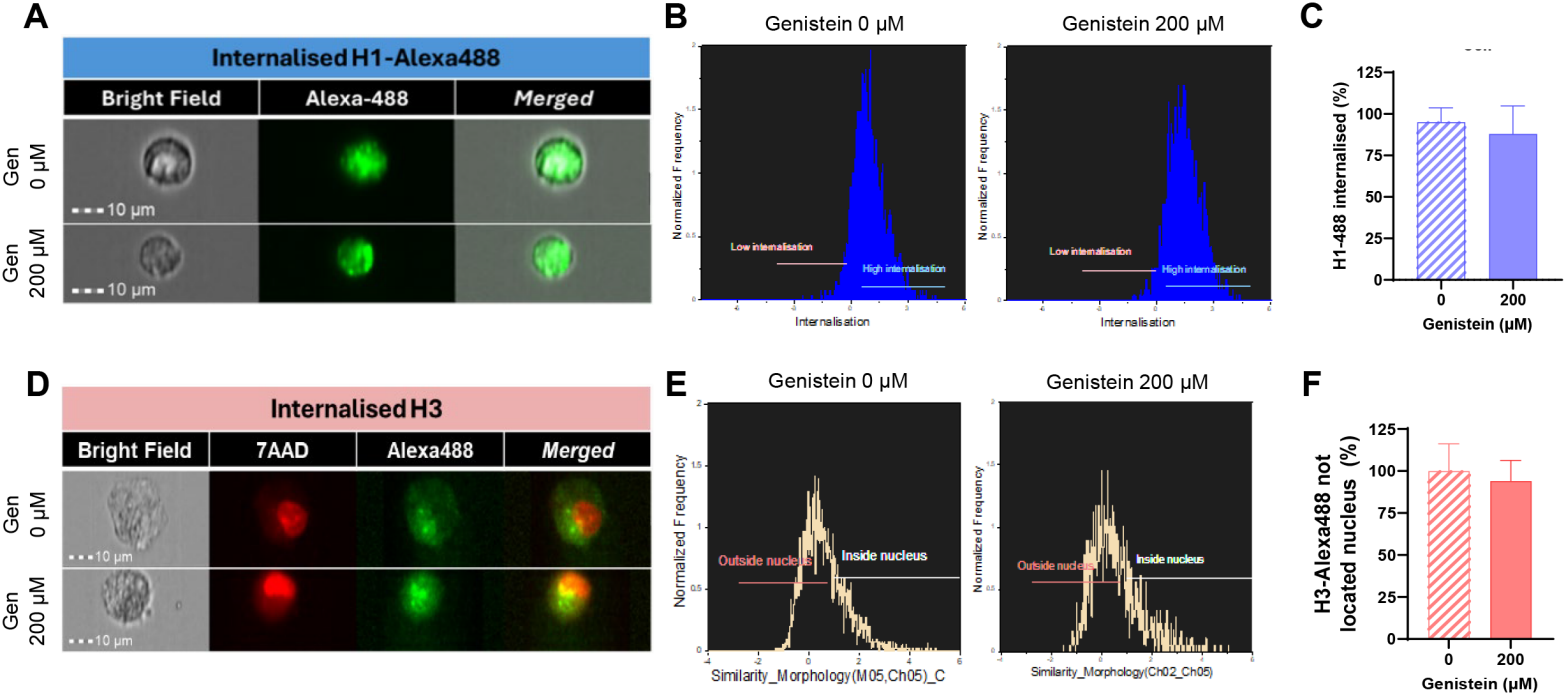
Effect of blocking caveolin-mediated endocytosis using genistein on the uptake of extracellular histones in HUVEC. Representative images of Alexa488-conjugated H1 **(A)** or H3-Alexa488 **(D)** internalization in HUVEC cells treated with genistein (0 or 200 μM) and 10 μg/ml H1-488 **(A)** or 50 μg/ml Core Histones (H2AB, H3, H4) **(C)** obtained with the INSPIRE software (ImagesStreamX Mark; Amnis-Merck Millipore). Red fluorescence corresponds to cellular nuclei stained with 7AAD and green fluorescence corresponds to histone H1-Alexa488 **(A)** or H3 histones stained with an Alexa488-conjugated primary antibody. Magnification 40X. 10 μm scale bars. **(B)** Histograms generated using the internalization wizard of the IDEAS software and **(E)** histograms resulting from the application of the internalization algorithm and the similarity feature to differentiate the green marker located in the nucleus from that not located in the nucleus. **(C, F)** Quantification of H1-Alexa488 internalization **(C)** or H3 located in the cytoplasm **(F)** of HUVEC using the internalization wizard provided by IDEAS software. Mean values are presented as bars, expressed as a percentage ± the standard deviation (n=3), with statistically significant differences considered when p<0.05 (T-test).

**Figure 6:**
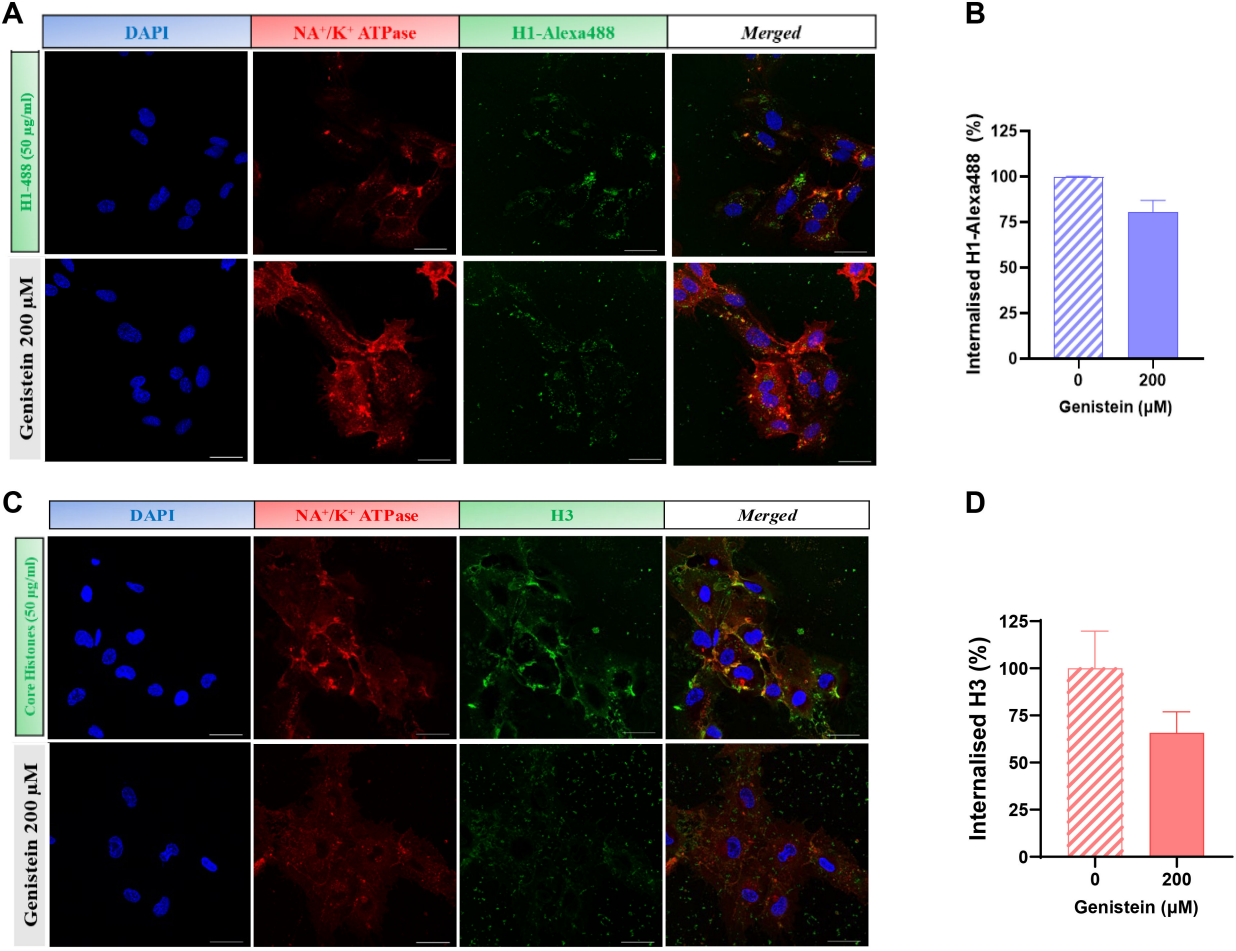
Confocal microscopy analysis of caveolin-mediated endocytosis inhibition with genistein. **(A, C)** Images of HUVEC treated with 50 μg/ml histone H1-488 **(A)** and 50. μg/ml **(C)** Core Histones (H2AB, H3 and H4). Blue fluorescence corresponds to cell nuclei by DAPI staining, red to Na+/K+ ATPase by binding to a secondary antibody conjugated to Texas Red fluorophore and the green one with the fluorescent histone H1-488 **(A)** or histone H3 by staining with a primary antibody conjugated to the fluorophore Alexa-488 **(C)**. In addition, a fourth column is shown with the fusion of the three channels used. Magnification 63X. 40μm scale bars. Quantification of the internalisation of histone H1-488 **(B)** or H3 **(D)** in HUVEC using the Cell Profiler 4.2.8 release software. Mean values are shown as bars ± the standard deviation of the percentage of internalisation (20 images per condition from 3 independent experiments). Statistically significant differences were considered with respect to the control (genistein 0 μm) when p<0.05 (T-test).

As observed in **Figure 2D**, treatment of HUVEC cells with 400 µg/mL of core histones for 4 hours is cytotoxic to the cells; however, when cells are incubated at 4°C, cell survival increases compared to normal conditions, indicating a beneficial effect of endocytosis blockade. The same was observed in the case of BVOs: when they were treated with 500 µg/mL of core histones survival is highly decreased, but when the organoids were pre-incubated at 4°C, survival was recovered (**Figure 2E**). To assess if specific endocytosis processes were involved in the internalization mechanisms of extracellular histones into HUVEC cells, we used imaging flow cytometry in the absence or presence of specific inhibitors of either clathrin- or caveolin-mediated endocytosis (see **Supplementary Figure S2** for an overview of the optimization process of endocytosis inhibition using reference controls in HUVEC). First, we used dynasore, a widely used inhibitor of dynamin (29, 30) to block clathrin-mediated endocytosis, resulting in a drastic reduction of histone internalization for both H1-488 (**Figures 3A–C**) and histone H3 (**Figure 3D–F**). This result was further confirmed by confocal microscopy analysis, using different concentrations of dynasore, and in both cases histone internalization was significantly reduced for both histone H1–488 and histone H3 (**Figure 4**); noteworthy, the same reduction of internal signal for histone H3 was registered in the case of HUVEC treatment with different concentrations of core histones (50 µg/mL and 200 µg/mL). Next, we performed the same experiments in the presence of genistein, a broad-spectrum tyrosine-kinase inhibitor which has been shown to block caveolin-mediated endocytosis (31, 32). In this case, the entry of histones H1-488 (**Figures 5A–C**) or H3 (**Figures 5D-F**) was not affected in a significant way, as further corroborated by confocal microscopy (**Figure 6**). Taken together, these results suggest that the cytotoxic effects mediated by extracellular histones require their entry into cells, and that internalization is mostly a clathrin-mediated endocytosis process.

### 2.3 Internalized histones H3 and H1 colocalize with the autophagy mediator LC3B in HUVEC

**Figure 7:**
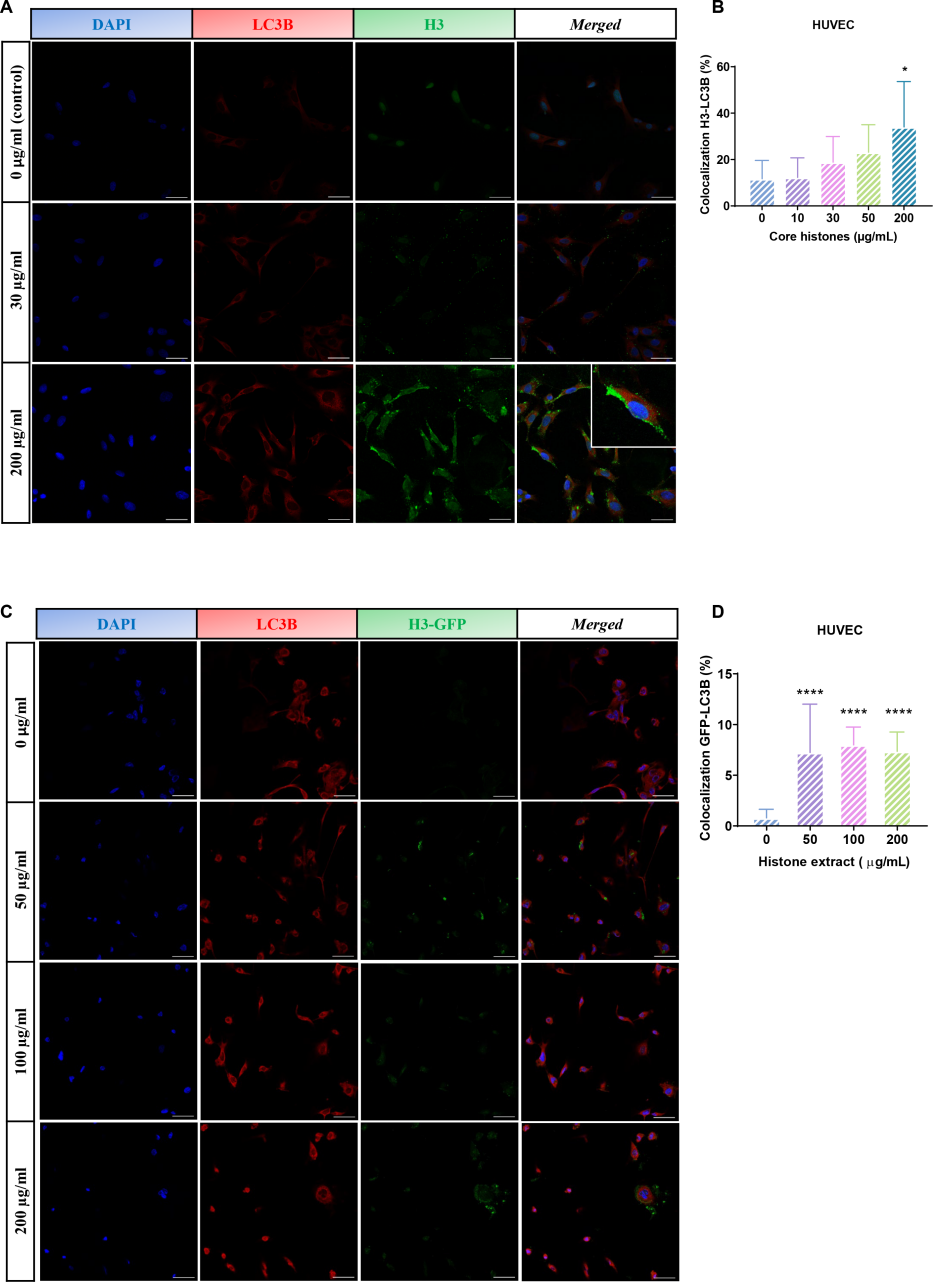
Analysis of colocalization between H3 histones or H3-GFP and autophagy marker LC3B. **(A, C)** Confocal representative images of HUVEC subjected to different concentrations of core histone treatment **(A)** or an extract of nuclear proteins enriched in histones **(C)**. Each row displays images from the same field of view. Blue fluorescence corresponds to cellular nuclei stained with DAPI, red fluorescence corresponds to LC3B protein labelled with a Texas Red-conjugated secondary antibody, and green fluorescence corresponds to H3 histones stained with an Alexa488-conjugated secondary antibody **(A)** or the fluorescence of H3-GFP **(C)**. Furthermore, a fourth column displays the merge of the three channels used (40 μm scale bars). The insert was obtained using a 4x zoom from the indicated image. **(B, D)** Quantification of colocalization performed with the Leica Application Suite X software (Leica Microsystems; Germany) (see section 5.4 Materials and Methods) between **(B)** histone H3 from core histone (Sigma) treatment or **(D)** GFP tag bound to histone H3 from treatment with an extract enriched in histones and LC3B protein. The mean values of colocalization percentage from 10 images per condition from 3 different experiments are represented as a bar graph, along with the standard deviation. Statistical significance was considered when p<0.05 compared to the control (0 μg/ml), calculated using a one-way ANOVA statistical test indicated by "*" for p<0.05 and "****" for p<0.0001.

Once the cytotoxic potential of core histones in HUVEC was evaluated, and we confirmed histone entry in both models, we aimed to study the subcellular location of histones upon entry and the relationship between exposure to extracellular histones and autophagy activation, as suggested by our previous studies (20). Subcellular localization studies of core histones were conducted using immunofluorescence, followed by quantification of the degree of colocalization between H3 histones and the autophagy protein LC3B. HUVEC were treated with core histones for 4 hours at various concentrations, followed by indirect immunofluorescence staining of H3 histone and LC3B (see **Supplementary Figure S3**). **Figure 7A** displays representative images for some of the analyzed conditions and **Figure 7B** shows the quantification of the colocalization. The colocalization analysis between green and red fluorescence was performed for each condition using the Leica Application Suite X software (Leica Microsystems; Germany). This software calculates the percentage of overlap between the two fluorophores per image (Leica Microsystems; Germany). Quantification revealed a dose-dependent increase in both fluorescent marks which co-localized inside cells with a very high significant increase observed at a core histone concentration of 200 µg/mL. In order to confirm that the previously obtained results were due to colocalization with extracellular histones used in the treatment rather than endogenous histones present in the cells, a parallel study on colocalization was conducted between a recombinant form of histone H3 fused with green fluorescent protein (H3-GFP) and the autophagic marker LC3B (**Figures 7C, D**). In this case, cultured HUVEC were treated with a histone extract enriched with H3-GFP obtained from HeLa cells transfected with the pIRES-Neo-H3.3-GFP plasmid to express the H3 protein tagged with GFP. As the histone extraction procedure led to a loss of GFP-fluorescence, an anti-GFP antibody was used. The histone extract was administered to cultured HUVEC at concentrations ranging from 50 to 200 µg/ml for 4 hours, and LC3B and GFP proteins were labelled with secondary antibodies conjugated to different fluorophores, while cellular nuclei were stained with DAPI.

**Figure 8:**
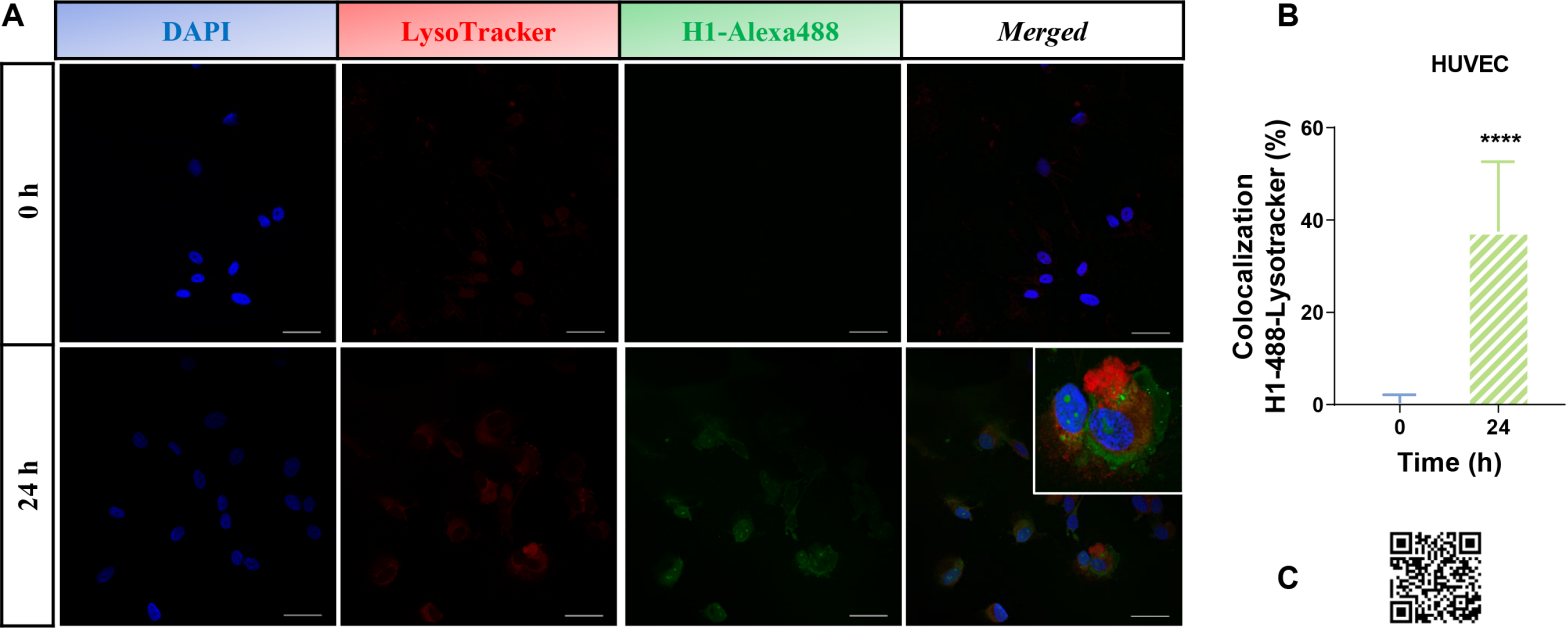
Analysis of colocalization between commercial histones H1-Alexa488 and LysoTracker Red DND-99. **(A)** Confocal microscopy images of HUVEC cells treated with histone H1-488 (50 μg/ml) for 24 hours. Blue fluorescence of each row shows cell nuclei by DAPI staining, red with the LysoTracker Red DND-99 acid organelle marker and green for histone H1-Alexa488, corresponding to the same visual field. In addition, a fourth column is shown with the fusion of the three channels used (40 μm scale bars). The insert was obtained using a 4x zoom from the indicated image. **(B)** Quantification of colocalization between LysoTracker Red DND 99 and histone H1-Alexa488 at the different incubation times with treatment, using the Leica Application Suite X software (Leica Microsystems; Germany) (see section 5.4 Materials and Methods). The bars represent the mean values ± the standard deviation of the percentage of colocalization (10 images per condition from 3 independent experiments). Statistically significant differences were considered with respect to the control (0 hours) and between the different conditions when p<0.05, calculated using T-test indicated by “****” when p<0.0001. **(C)** QR leads to a 24-hour time-lapse video in which increasing orange signal indicates more accumulation of H1-488 (green) at acidic organelles (red).

**Figure 3A, D** shows a significant dose-dependent increase in between the LC3B marker and the histone- H3-GFP protein, confirming the previous results. Also, HUVEC cells treated with histone H1–488 at a concentration of 50 µg/ml for 24 hours were studied for colocalization between H1-488 (green fluorescence) and the acid organelle marker Lysotracker Red DND99 (red fluorescence) at different time points, using indirect immunofluorescence and confocal microscopy (**Figures 8A, B**). A live cell image video can be viewed via the QR code (**Figure 8C**). Quantification revealed a significant increase in colocalization between both proteins when the treatment was extended to 24 hours.

**Figure 9:**
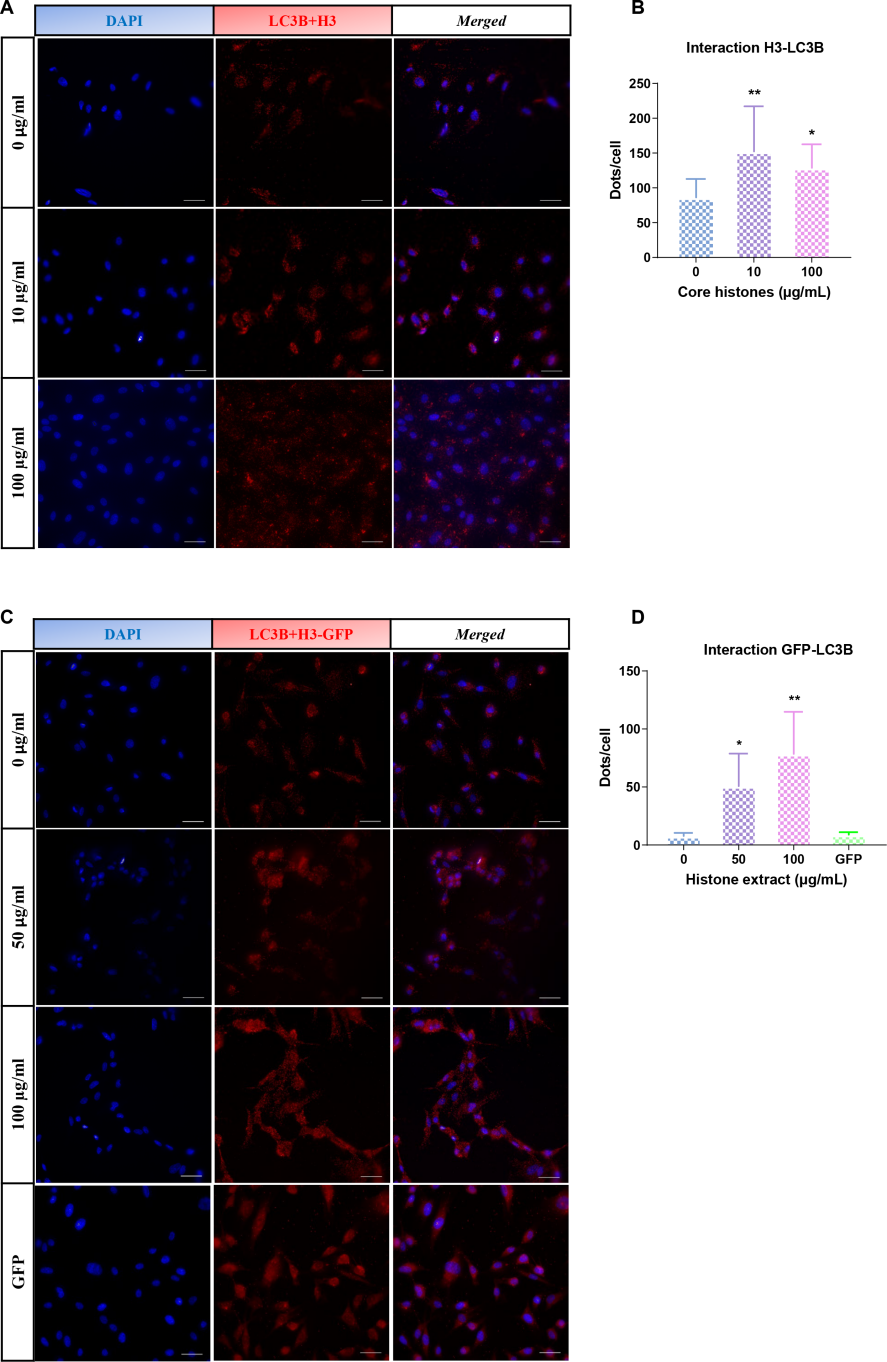
Analysis of interaction between commercial H3 histones or H3-GFP extract and LC3B using PLA. **(A, C)** Fluorescence microscopy images of HUVEC after treatment with the curve of increasing concentrations of purified commercial histones (H2AB H3 and H4) **(A)** or histone H3-GFP extract **(C)**. The blue fluorescence corresponds to cell nuclei by DAPI staining, while the red fluorescence corresponds to interactions between histone H3 **(A)** or GFP tag bound to histone H3 **(C)** and LC3B protein (due to their physical proximity, which allows ligase binding of the secondary antibody-bound nucleotide tails and their amplification by polymerases with fluorescent nucleotides). The third column represents the fusion of the two channels used. Also, a GFP (vehicle) control is represented (40 μm scale bars). **(B, D)** Quantification of the interactions between H3 histone **(B)** or GFP tag **(D)** and LC3B protein per cell. The mean values of the interactions produced in 80 cells per condition from three different experiments are represented as a bar graph, along with the standard deviation. Statistical significance was considered when p<0.05 compared to the control (0 μg/ml), calculated using a one-way ANOVA statistical test indicated by "*" for p<0.05 and “**” for p< 0.01.

To further confirm a possible interaction between LC3B and exogenous histone H3, a proximity ligation assay (PLA) was performed. HUVEC cells were treated with core histones at concentrations between 10 and 100 µg/ml (**Figures 9A, B**), and with H3-GFP histone extract at concentrations between 50 and 100 µg/ml (**Figures 9C, D**) for 4 hours. In at least 80 cells from each condition, fluorescent red dots derived from the interaction between LC3 and H3 (**Figure 9B**) or H3-GFP (**Figure 9D**) were quantified. In the experiment with core histones, quantification revealed a significant increase in the colocalization between histone H3 and LC3B at concentrations of 10 and 100 µg/ml and the same was observed in the case of the treatment with histone extract containing H3-GFP.

These findings indicate that histones added to HUVEC are capable of entering cells and, subsequently, they co-localize with LC3B, suggesting a link between the processes of extracellular histones-mediated cytotoxicity and LC3-mediated pathways.

### 2.4 Increase in LC3B expression after exposition of BVOs to extracellular histones H3 and H1 is prevented by endocytosis blockade

**Figure 10:**
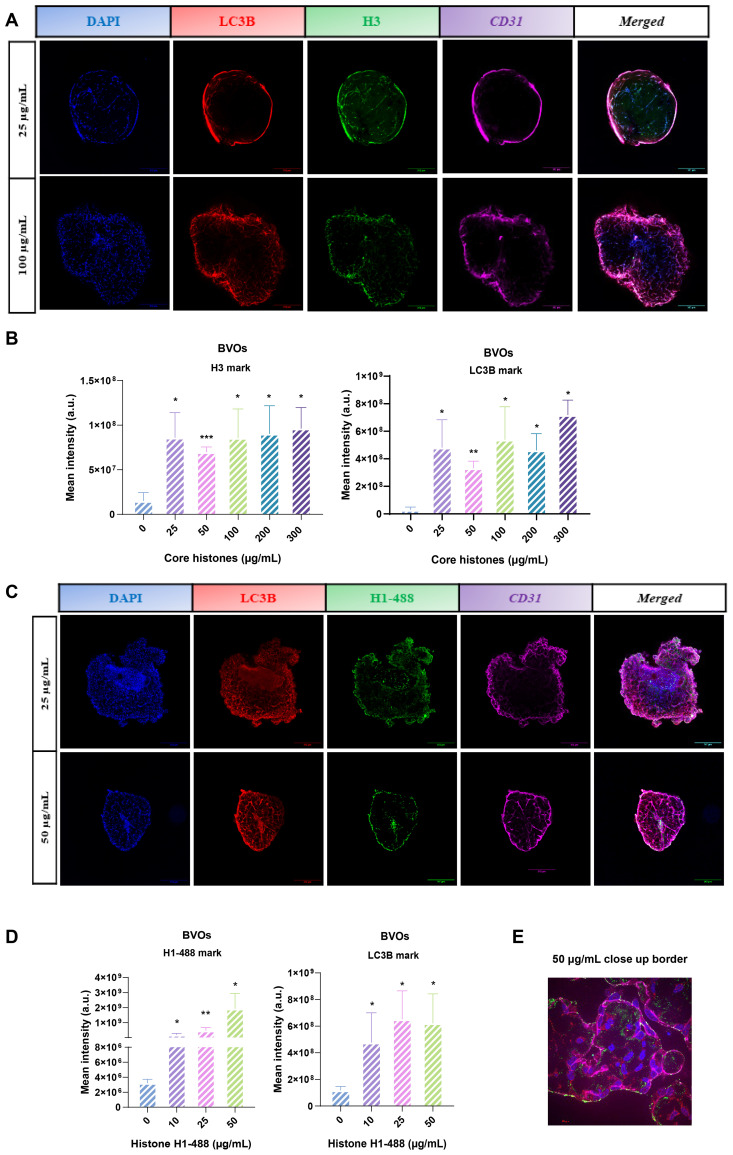
Analysis of histone H3 or H1-Alexa488 and LC3B on blood vessel organoids (BVOs) treated with core histones or H1-Alexa488. **(A, C)** Confocal microscopy images of BVOs that were subjected to treatment with several concentrations of core histones for 4 hours or H1-488 for 24 hours, respectively. The blue fluorescence of each row shows cell nuclei by DAPI staining, red fluorescence corresponds to LC3B autophagy marker protein, the green one to histone H3 and purple one to CD31 endothelial cell marker, corresponding to the same visual field in the middle section of the organoid. In addition, a fifth column is shown in **(C)** with the fusion of the four channels. Magnification 10X. Scale bars 312 μm. All slices deep from a z-stack of a whole organoid scan (25 ug/mL 34 z plane, 50 ug/mL 23 z plane). **(B, D)** Quantification of the mean fluorescence intensity of green channel (H3 in B or H1-488 in D) and red channel (LC3B) by ImageJ software. **(E)** Images from the border of the organoid treated with 50 μg/mL of histone H1-488. Scale bar 31 μm. The slice deep from a z-stack of a whole organoid scanned and corresponds to the 14 z plane. The histogram’s bars represent the mean values ± the standard deviation of the percentage of colocalization of each condition (n=3). Statistically significant differences were considered with respect to the control (0 μg/mL) when p<0.05, calculated using ANOVA one-way indicated by “*” when p<0.05, “**” from p<0.01 and “***” when p<0.001.

After observing that extracellular histones also exhibit cytotoxic potential in the vascular organoid model, and that they colocalize with LC3B in HUVEC cells, we aimed to investigate the possible interaction between LC3B-mediated signaling and extracellular histones in BVOs. To accomplish this, once the organoids were fully differentiated and mature, they were treated with core histones for 4 hours (**Figures 10A, B***)* or with histone H1-488 (**Figures 10C–E***)* for 24 hours, at different concentrations with a minimum of five organoids per condition. Subsequently, the organoids were collected and processed for immunofluorescence staining using LC3B, H3, CD31 and, in the case of H1 from its fluorescent tag. Confocal microscopy images of at least three organoids per condition were captured. The average fluorescence intensity of the green signal corresponding to indirect immunofluorescence labelling of histone H3, and the red signal corresponding to the autophagic flux protein LC3B (**Figure 10B**) or the intrinsic green fluorescence of H1–488 and indirect immunofluorescence labelling of LC3B (**Figure 10D**) were quantified using ImageJ, measuring the average fluorescence of each image and substracting the background. We show that, even at low doses, the signal corresponding to histone H3 no longer coincides with the blue fluorescence (DAPI-DNA staining) of the nuclei, but instead shows a more homogeneous distribution throughout the organoid, particularly at the organoid’s edge and center. Furthermore, treatment with the histones leads to an onset of green fluorescence which is maintained throughout all tested concentrations. The signal of LC3B is also predominantly distributed at the organoid’s edge, with the fluorescence intensity showing the same pattern - increasing when exposed to core histone treatment. For histone H1–488 a similar trend was observed: histone fluorescence was predominantly located at the center and the edge of the organoid while LC3 fluorescence was more evenly distributed with an increased accumulation at the edge (**Figure 10C**), whereas the zoom (**Figure 10E**) shows green fluorescence predominantly located inside the vessels formed by endothelial cells. Quantification of the fluorescence intensity (**Figure 10D**) showed an increase in both green and red signal in a dose-dependent manner. Based on these observations, we believe that there is a link between extracellular histones and LC3-mediated pathways in BVOs.

**Figure 11:**
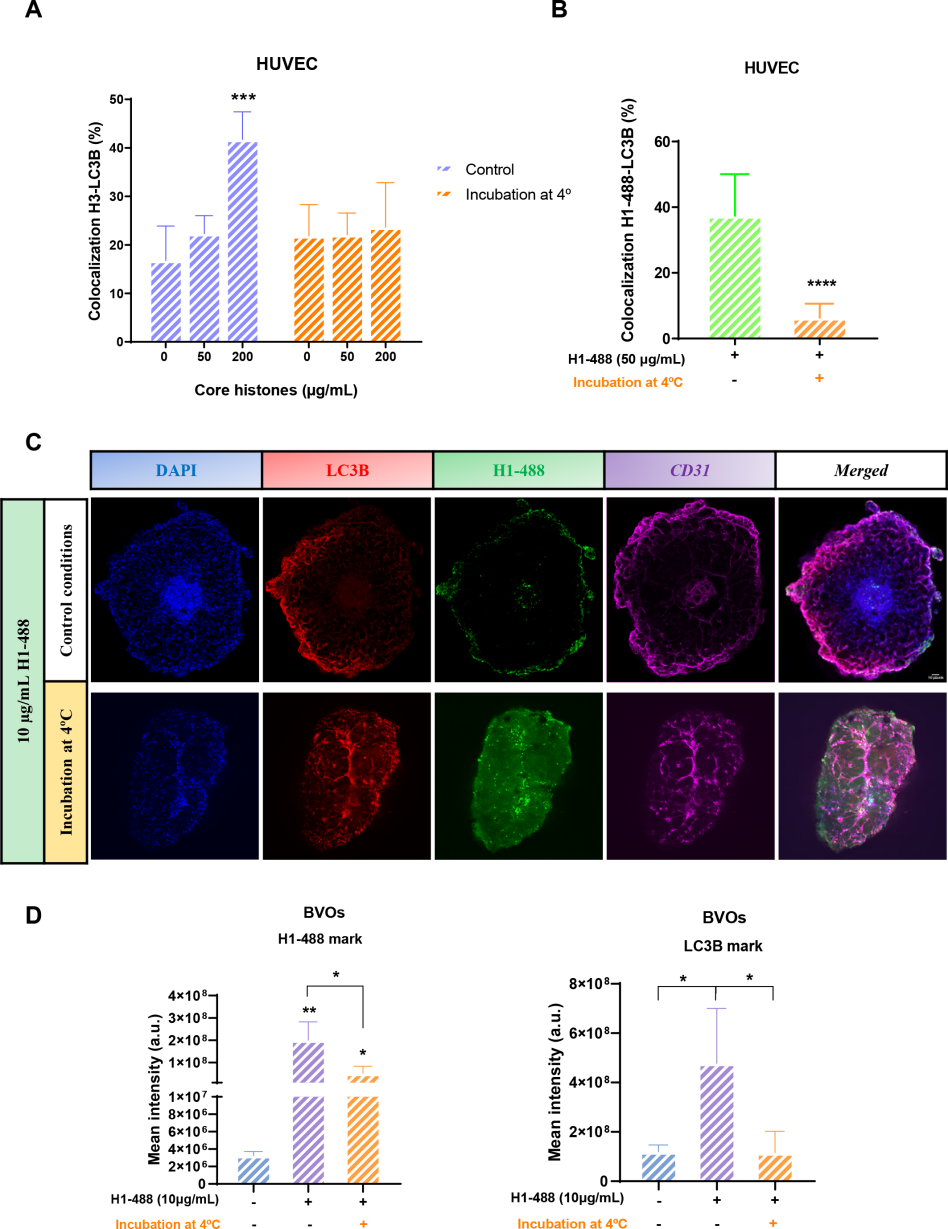
Effect of blocking endocytosis on colocalization between histone H3 or H1-488 and LC3B. Quantification of colocalization between histone H3 **(A)** or H1-488 **(B)** and LC3B protein under control or endocytosis blockade conditions performed using the TCS SP8 X confocal microscope software (Leica Microsystems; Germany). **(C)** Confocal microscopy images of BVOs treated with histone H1-488 at 10 μg/ml under control and endocytosis-blocking conditions. Each row shows images from the same field of view, with the blue fluorescence of each row shows cell nuclei by DAPI staining, red fluorescence corresponds to LC3B autophagy marker protein, the green one to histone H1-488 and purple one to CD31 endothelial cell marker, corresponding to the same visual field in the middle section of the organoid. In addition, a fifth column is shown with the fusion of the fourth channels used. Magnification, 10X. Scale bars 312 μm. Both slices deep from a z-stack of a whole organoid scan (control condition 46 z plane and incubation at 4ºC 21 z plane. **(D)** Quantification of the mean fluorescence intensity of green channel (H1-488) and red channel (LC3B) by ImageJ. The bars represent the mean values ± the standard deviation of the mean intensity value of fluorescence of each condition (n=3). Statistically significant differences were considered with respect to the control when p<0.05, calculated using ANOVA one-way indicated by “*” when p<0.05, “**” when p<0.01, “****” when p<0.0001 and n.s. as non-significant.

Based on the existing evidence in the literature, which suggests that nucleosome internalization into cells occurs through endocytosis (24), and our previous results in HUVEC, we wanted to assess if incubation of BVOs at 4°C prevented the aforementioned changes in both the internalization of histones and the expression of LC3B through BVOs. To achieve that, HUVEC were treated with core histones and incubated on ice for 4 hours. Nuclei were stained with DAPI, while histone H3 and LC3B protein were labelled by indirect immunofluorescence staining. As shown in **Figure 11A**, there was no significant increase in colocalization between histone H3 and LC3B, as already confirmed in the previous assays. To further check the observed effect, we repeated the experiment with histone H1–488 and could confirm that incubation at 4°C prevents an increase of colocalization between LC3 and extracellular histones (**Figure 11B**).

Finally, and to further verify our results with HUVEC, we incubated BVOs at 4°C and treated them with H1-488. **Figure 11C** shows that when histone entry is blocked, the H1–488 histone marker is observed on the surface of the organoid but not inside the cells. The green dots were clustered around the organoid surface, indicating that histone H1–488 fails to penetrate the organoid surface and consequentially the cells. This explains why the fluorescence intensity of the histone marker (**Figure 11D**) is higher compared to the non-histone treatment condition, but significantly lower than in the condition where histone treatment occurs under normal conditions, allowing the internalization of histones. Regarding LC3B (**Figure 11D**), when histone entry is blocked, no increase in LC3B fluorescence intensity was observed, remaining similar to the control without histone addition, suggesting that an endocytosis-dependent process is necessary to induce the increase in LC3B intensity. Based on these findings, we can propose that, similar to HUVEC cultures, histones are internalized into BVOs and that this internalization is necessary for histones to exert their cytotoxic effects on the organoids.

## 3 Discussion

The pathophysiological effects of diseases like sepsis must be understood under the light of the interactions among DAMPs, PAMPs, the different cell lines circulating in the bloodstream and, importantly, the endothelial barrier. In this heterogenous and complex landscape, it is critical to understand the relationships among extracellular histones, cellular entry pathways, and subsequent cellular responses. Herein we present a thorough analysis of these links by studying the cytotoxic potential of extracellular histones, providing insights into their mechanisms of entry into cells, their subcellular distribution, and their interaction with LC3B in both HUVEC and BVOs.

The initial focus of our study was to assess the cytotoxicity induced by extracellular core histones (H2A, H2B, H3, and H4) and histone H1–488 in HUVEC and BVOs. Our results align with existing literature demonstrating dose-dependent cytotoxicity of extracellular histones in different models and tissues, with different effects depending on experimental conditions and cell types that range between 10 – 500 µg/mL (20, 33–36). Interestingly, our results showed that high concentrations of core histones were needed to observe a significant decrease in cell survival and that the BVOs exhibited slightly increased resistance to histone-induced toxicity, possibly attributed to their 3D structure and diverse cell composition, highlighting the importance of using physiologically relevant models (16, 17). It should be noted that our experimental approach is not representing the full spectrum of plasma concentrations of extracellular histones in sepsis patients, which have been quantified by our group and others to values ranging from 10–5000 ng/mL (12, 37–39). Thus, the specific conditions and concentrations of endothelial cells exposed to circulating histones in the plasma of septic patients warrants further research. However, to our knowledge, this is the first documentation of the cytotoxic effects of extracellular histones in a complex, human pluricellular cell model such as BVOs, opening interesting avenues for research on the pathophysiological mechanisms related to sepsis. These findings contribute to our understanding of the detrimental effects of extracellular histones, especially in the context of sepsis, and highlight the relevance of employing physiologically relevant models to study complex diseases.

Previous studies have investigated the ability of histones to penetrate cells (21), but few have elucidated the underlying mechanisms. Expanding upon the previously observed cytotoxicity, we investigated the process of histone internalization into BVOs and HUVEC. Our results clearly demonstrate that one important determinant of histone-mediated cytotoxicity is histone entry into cells; histone internalization was successfully stopped by blocking entry through incubation of cells and BVOs at low temperatures, suggesting the participation of endocytosis-related absorption processes. This aligns with existing literature suggesting endocytosis as a crucial route for histones (40) and nucleosome internalization (24). In fact, Wang et al. (24), demonstrated that nucleosomes initially interact with the cell membrane through nonspecific non-electrostatic interactions with the positively charged histone tails, and subsequently enter the cells through clathrin- or caveolin-dependent endocytosis. Under our experimental conditions, only inhibition of clathrin-mediated endocytosis prevented histones from entering HUVEC cells; this discrepancy with the results from Wang and collaborators may be due to the different cells and reagents used, since caveolin-mediated internalization might be necessary to engulf the larger and more structurally complex nucleosomes (30) as compared to single H1 or core histones which we used in our study; nonetheless, the details underlying the specificity within these processes warrants further study.

**Figure 12:**
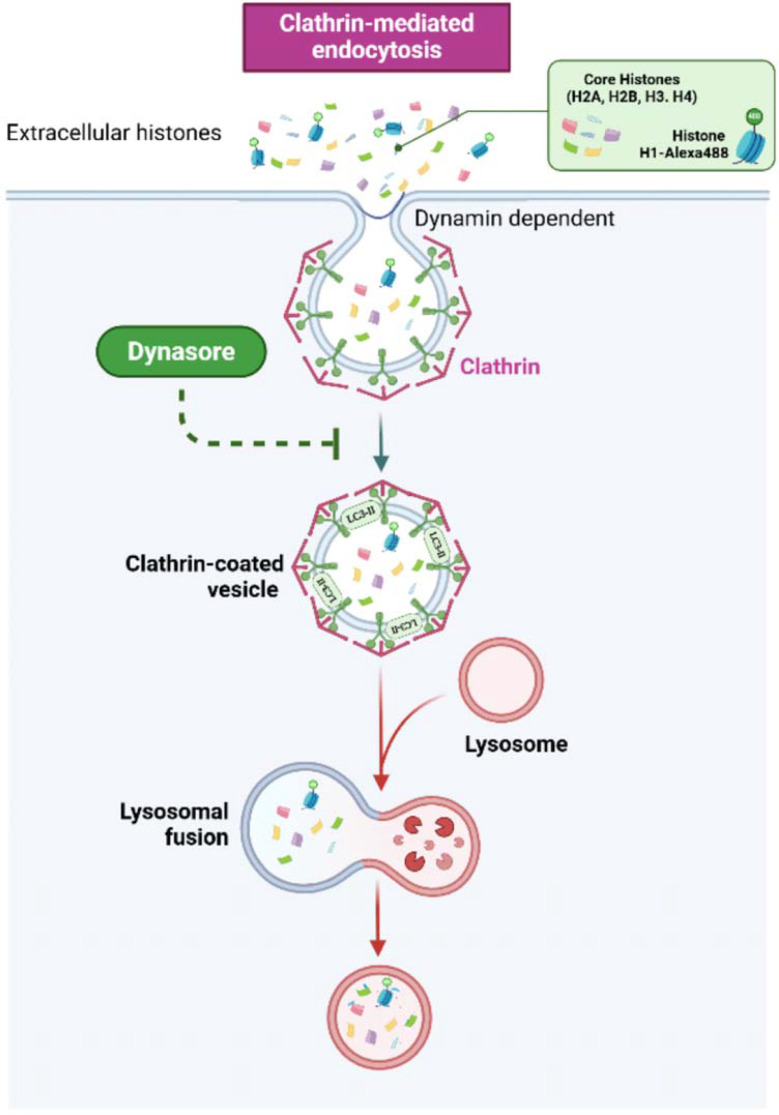
Suggested mechanism of histone entry into cells via non-canonical autophagy. Extracellular histones interaction with the plasma membrane of HUVEC cells might lead to internalization through clathrin-mediated endocytosis, after the endocytosed histones are directed to LC3-positive vesicles, suggesting a link with the so-calledd LC3-mediated non-canonical autophagy pathway. LC3-II: Light chain protein 3B.

After demonstrating that entry of histones takes place both in HUVEC and BVOs, we investigated the subcellular location of internalized histones and their possible interactions with the autophagy pathway. In HUVEC, colocalization between histone H3 and the autophagic marker LC3B increased in a dose-dependent manner according to immunofluorescence and subsequent colocalization analysis. Our proximity ligation experiments corroborated these findings and demonstrated a substantial interaction between histone H3 and LC3B. Significantly, these putative interactions were further confirmed by extending our findings to histone H1-488, which suggests that both extracellular histones H3 and H1 follow similar pathways when they interact with HUVEC. The intricate relationship between these histones and LC3B points towards a potential role of autophagy in response to histone-induced cellular stress. Our studies with HUVEC revealed the absence of increased colocalization between the LC3B protein and extracellular histones when the entry of histones was blocked, supporting the idea that endocytosis is a possible route of histone entry into cells, an effect that was also observed using H1-488. In addition, we demonstrated that the entry of histones by endocytosis mechanisms was necessary to induce the acute cytotoxic effects of extracellular histones. Taken together, these processes could be related to the concept of non-canonical autophagy, and particularly, point to LC3B-associated endocytosis (LANDO) (41, 42) (**Figure 12**). During this process, components of the autophagic machinery are utilized to conjugate LC3 protein with membranes of clathrin-positive endosomes. Currently, this type of endocytosis has been associated with the recycling of receptors such as TREM-1 or TLR-4 in microglia (41), highlighting that in the context of sepsis, these pattern recognition receptors are involved in the development of the disease’s pathogenesis, but this possibility should be further investigated. Another evidence that supports this hypothesis is the fact that endothelial cells have been shown to activate non canonical autophagy during inflammation (43). The possibility of non-canonical autophagy-mediated delivery of extracellular histones to autophagosomes in endothelial cells and complex models such as blood vessel organoids holds promising implications for understanding the inflammatory signaling cascade associated with sepsis. Further experiments are warranted to validate and explore this intriguing research avenue; however, our research adds significant understanding to the complex function of extracellular histones in sepsis and vascular dysfunction. The possible protective function of autophagy in reducing histone-induced cytotoxicity prompts inquiries regarding the wider consequences of regulating autophagy in sepsis and associated illnesses. To fully understand how autophagy interacts with histone-induced changes and investigate the possibility of autophagy modulation as a therapeutic target, more research is necessary; nonetheless, a better understanding of the molecular implications of the contact between extracellular histones, released during inflammatory response to infection, and endothelial cells, could potentially improve their use as biomarkers of disease severity and progression, since the relationship between extracellular histone levels and vascular or coagulation alterations has been documented extensively (39).

To sum up, in this work we offer a thorough investigation into the lethal potential of extracellular histones in vascular models, illuminating the complex interactions among autophagic responses, subcellular localization, and histone entry pathways. Deciphering these intricate relationships paves the way for subsequent investigations focused on creating tailored treatment plans to mitigate the deleterious consequences of extracellular histones in sepsis and associated vascular diseases. Further investigation into the molecular mechanisms underlying autophagy pathway modification and histone-induced cytotoxicity holds potential to advance our comprehension of these intricate biological processes and enhance clinical outcomes for patients with sepsis.

## 4 Materials and methods

### 4.1 Cell culture

#### 4.1.1 2D culture

HUVEC were purchased from Cultek (Lonza, Cultek, Spain). They are a pool of primary cells from several donors to avoid donor variability. The surface of plates and flasks were covered with 1% w/v gelatine (Sigma-Aldrich, United Kingdom). They were cultured on specific growing medium EGM-2 (Endothelial Growth Medium 2) (Lonza, Cultek, Spain) supplemented with 5% v/v of FBS, 0,04% v/v of hydrocortisone, 0,4% v/v of hFG-B (Human Basic Fibroblastic Growth Factor), 0,1% v/v of VEGF (Vascular Endothelial Growth Factor), 0,1% v/v of R3-IGF-1 (Long Arg3 Insulin-Like Growth Factor- 1), 0,1% v/v of ascorbic acid, 0,1% v/v hEGF (Human Epidermal Growth Factor), 0,1% v/v of GA-1000 (Gentamicin sulfate/Amphotericin), 0,1% v/v of heparin (Lonza, Cultek, Spain) and 1% v/v P/S.

Cells were incubated at 37°C and 5% v/v CO2. The passage of cells was done when the cell culture reached an 80% confluency, removing the media, washing cells with phosphate buffer saline (PBS) and detaching them with EDTA (Ethylenediaminetetraacetic acid) trypsin (Sigma-Aldrich, United Kingdom). All cell types were tested for mycoplasma regularly.

#### 4.1.2 Blood vessel organoids

Blood vessel organoids were obtained though the differentiation of either H9 embryonic stem cells or NC8 human iPSC (induced pluripotent stem cells). Stem cells were grown on Matrigel covered plates with Essential 8 medium (Thermo Scientific, USA). After thawing, cells were passaged at least twice before initiating a differentiation (16, 17).

#### 4.1.3 Treatments

Several types of extracellular histones were used: “core histones” consists of a mixture of H2A, H2B, H3 and H4 histones purified from calf thymus (Sigma-Aldrich, United Kingdom); histone H1-Alexa488 consist of purified H1 from calf thymus conjugated to a 488-fluorescent tag (Thermo Scientific, USA); Histone H3-GFP (Histone 3-Green Fluorescent Protein) consists of a nuclear extract enriched with a recombinant form of histone H3 with a GFP tag (see plasmid and transfection details in next section). The extracellular histones were added directly to the culture medium and incubated at 37°C during the specific time depending on the experiment.

For blocking macropinocytosis, cells were incubated at 4°C for 15 min prior any histone treatment. Then, histone treatment was performed, and the cells were returned to ice for the entire duration of the specific treatment. For clathrin and caveolin-mediated endocytosis inhibition, 1h serum-starved HUVEC (Krebs-Henseleit medium; Thermo Scientific, USA, supplemented with 2.1 g/L of sodium bicarbonate and 0.373 g/L of calcium chloride) were incubated during 1 h with vehicle (DMSO) or different concentrations of dynasore (Merck-Sigma, Germany) and genistein (Merck-Sigma, Germany). Then the specific histone treatments were applied to the cells.

### 4.2 Transfection

HeLa cells were transfected with 2.5 µg of pIRES-Neo-H3.3-GFP plasmid kindly provided by Dr. Sandra B. Hake (Institute for Genetics, University of Giessen, Germany) using 1 mg/ml polyethyleneimine (PEI) (Polysciences, Germany) in Opti-Mem (Gibco, Thermo Scientific, USA). HeLa cells were incubated for 24h with the mixture of PEI and plasmid and afterwards medium was replaced by DMEM media. Transfected cells were analyzed by confocal microscopy (Zeiss, USA) maintaining cells at 37°C and 5% v/v CO2, using bright field and argon laser at 488 nm.

### 4.3 Histone extraction

The histone extraction is a cell fractionation protocol in which the nuclear fraction predominates while histone enrichment is favored. Here, we took advantage of the fact that histones are soluble in sulfuric acid and that they precipitate in the presence of TCA (trichloroacetic acid). Hela cells were seeded until they reached 80% confluency, medium was removed, and cells were pelleted. Pelleted cells were resuspended with 1mL hypotonic Lysis Buffer (Tris-HCl 10 mM (pH = 8), KCl 1 mM y MgCl2 1,5 mM, orthovanadate (10 µL/mL) and protease inhibitor cocktail (Sigma-Aldrich, United Kingdom), and left for 30 min in the rotatory shaker at 4°C. After that, the tubes were centrifuged for 10 min at 12750 rcf at 4°C and the supernatant was discarded, and the pellet was resuspended with 320 µl of H_2_SO_4_ 0,5 N and left overnight in the rotatory shaker at 4°C. After the incubation, cells were centrifuged for 10 min at 16750 rcf at 4°C, the supernatant was collected and transferred to a new tube and 132 µl of TCA were added. Tubes were incubated on ice for 30 min and afterwards they were centrifuged for 10 min at 16750 rcf at 4°C. The supernatant was discarded, and the pellet was resuspended with 0.5 mL of cold acetone and the tubes were centrifugated 10 min at 16750 rcf at 4°C. The last step needs to be repeated twice. Finally, the pellets were dried for 5 min and resuspended with 50 µl of deionized water. Histone quantification was performed using BCA method as previously described.

### 4.4 Antibodies

Antibodies used in the present work are listed in (**Table 1**).

**Table 1:** Primary antibodies used.

Antibody	Reference	Technique and dilution used
LC3B-II	L7543 Sigma-Aldrich United Kingdom	IF 1:1000 or 1:200 (BVOs) PLA 1:1000
GFP	9996 Santa Cruz Biotechnology, USA	IF 1:200 PLA 1:200
H3	06–755 Merck Millipore, Germany	IF 1:1000 or 1:500 (BVOs) PLA 1:1000
H3-Alexa488	Ab203850, Abcam, UK	IF 1:1200 IFC: 1200
Na^+^/K^+^ ATPase	Ab76020 Abcam, UK	IF 1:1000
CD31 (mouse)	Ab9498 Abcam, UK	IF 1:100 (BVOs)
CD31 (sheep)	#AF806 R&D Systems, USA	IF: 1:200 (BVOs)

IF, Immunofluorescence; PLA, Proximity Ligation Assay; BVOs, Blood Vessel Organoids; IFC, Imaging Flow Cytometry.

### 4.5 Image techniques

#### 4.5.1 Immunofluorescence

HUVEC were seeded on 13 mm diameter glass crystals inside twenty-four well plates until they reached adequate confluency and the corresponding treatments were made. Afterwards, medium was removed, and cells were rinsed with 1x PBS. For fixation and permeabilization 300 µL of methanol was added to the cells and they were incubated for 15 minutes at -20°C. Then, methanol was removed, cells were washed three times with PBS-Tween 0.1% for 5 minutes at room temperature in agitation and they were blocked with 10% FBS in PBS-Triton 0.1%. Cells were incubated with blocking solution for at least 1h at room temperature and after that time, blocking solution was removed and primary antibody (see **Table 1**) diluted in blocking solution was added and incubated overnight at 4°C. The next day, primary antibody was removed, the cells were washed three times with PBS for 5 minutes and they were incubated with corresponding secondary antibodies (Alexa-Fluor-488-conjugated goat anti-mouse; A11029, Invitrogen, USA) and Alexa-Fluor-Texas Red-conjugated goat anti-rabbit; T862, Invitrogen USA) diluted 1:2000 in blocking solution for 1h at room temperature. Finally, the crystals were mounted on slides with DAPI-Fluoromount-G^®^ mounting medium (Southern Biotech; USA) for nuclei detection and to later be observed under a confocal- or epifluorescence microscope.

For some experiments, instead of using a primary antibody to mark lysosomes (LC3B-II), a fluorescent probe was used. Briefly, after the respective treatment was made to the cells, the medium was removed and 75nM of Lysotracker Red DND-99 (Invitrogen, USA) was added to the cell culture medium and incubated for 30 minutes at 37°C. Afterwards, the medium with Lysotracker was removed, and the immunofluorescence protocol was followed as explained above.

Blood vessel organoids were individually cultured in 96-well ultra-low attachment plates and the corresponding treatments were made (five organoids were used per condition). Afterwards, organoids were collected into 2 mL tubes and the medium was removed carefully. For fixation 1 mL of 4% PFA (paraformaldehyde) was added to the organoids, and they were incubated for 2 hours at room temperature. Then, PFA was removed, organoids were washed twice with PBS for 15 minutes at room temperature in agitation and they were simultaneously permeabilized and blocked with 1 ml of blocking solution (3% FBS, 1% BSA, 0.5% Triton X-100 and 0.1% Sodium deoxycholate in PBS) and incubated for 2 hours at room temperature. After that time, blocking solution was removed and primary antibody (see **Table 1**) diluted in blocking solution was added and incubated overnight at 4°C. The next day, primary antibodies were removed, the organoids were washed three times with PBS-Tween20 0.05% for 15 minutes and they were incubated with DAPI (Invitrogen, USA) diluted 1:750 in blocking solution and the corresponding secondary antibodies (Alexa-Fluor-488-conjugated goat anti-mouse (Invitrogen, USA), Alexa-Fluor-555-conjugated goat anti-sheep (Invitrogen, USA), Alexa-Fluor-647-conjugated goat anti-rabbit (Invitrogen, USA) and Alexa-Fluor-555-conjugated goat anti-mouse diluted 1:250 in blocking solution for 2h at room temperature. Then, the secondary antibodies were removed, and organoids were washed twice with PBS-T and once with PBS. After that organoids were incubated with 300 µL of RapiClear CS solution (Sunjin Lab, Taiwan) for 1 h at room temperature for tissue cleaning. Following, organoids were mounted between two coverslips separated by a 0,05 mm Spacer and sealed with RapiClear CS gel (both from Sunjin Lab, Taiwan). Finally, organoids were observed under a confocal microscope. To create visual representations of vascular networks and complete blood vessel organoids, we utilized a confocal microscope equipped with a 10x lens.

#### 4.5.2 Proximity ligation assay

Proximity Ligation Assay (PLA) is a technique used to study protein-protein interactions by providing a single fluorescent signal for each interaction event (44). HUVEC were seeded into eight well plates with glass bottom (IBIDI GmbH, Germany) until they reached 80% confluency, and the appropriate treatments were made. After that, medium was removed, cells were rinsed with 1x PBS, and they were fixed and permeabilized with methanol for 15 min at -20°C. Then cells were washed three times with 1x PBS for 5 minutes and they were blocked with 100 µL of Duolink^®^ II Blocking Solution (Sigma-Aldrich, United Kingdom) for 1h at 37°C. After the incubation, the blocking solution was removed, and two specific primary antibodies (**Table 1**) diluted with 1x Duolink^®^ II Antibody Diluent (Sigma-Aldrich, United Kingdom) were added and incubated overnight at 4°C. The day after, the antibodies were removed, cells were washed twice with TBS-Tween 0,01% and cells were incubated with the Duolink^®^*in situ* PLA^®^ Anti-rabbit PLUS (Sigma-Aldrich, UK, DUO92002) and Anti-mouse MINUS (Sigma-Aldrich, UK, DUO092004) for 1h at 37°C. Then, the probes were removed, and the cells were washed twice with TBS-T 0.01% for 5 min in agitation. Ligation and amplification steps were performed using the *in-situ* detection reagent kit Duolink^®^ Red (Sigma-Aldrich, UK, DUO92008-100RXN). For ligation, DuoLink^®^ Ligation Stock was diluted 1:5 with ultrapure water and then the ligase was added to the mix in a dilution 1:40. After, 100 µL of the ligation solution were added to the cells, incubated for 30 min at 37°C. Then the ligation solution was removed, and cells were rinsed twice with TBS-T 0.01%. For amplification step reagents and cells must be protected from light. DuoLink^®^ amplification stock was diluted 1:5 with ultrapure water and then the polymerase was added to the mix in a dilution 1:80. After, 100 µL of the amplification solution were added to the cells, incubated for 100 min at 37°C. After, polymerase solution was eliminated and cells were washed once with 1x SSC buffer for 2 min and then with 0,01x SSC buffer for 2 min. Finally, for nuclei detection DAPI Fluoromount-G^®^ (Southern Biotech, USA) was added. All the incubations done at 37°C were made in a humid chamber and all the washing steps were done in agitation. The dots were counted manually, and the results were expressed as the number of interactions (dots) per number of nuclei in each field. The interactions per cell of each condition are expressed as a percentage of the control condition.

#### 4.5.3 Confocal microscopy

The slides with the immunofluorescence crystals were placed in a TCS SP8 X confocal microscope equipped with a white laser and 4 spectral detectors, one of them hybrid Leica HyD (Leica Microsystems, Germany), and observed at 40,000x or 63,000x magnification in immersion oil. A minimum of ten images of each condition were captured when the sample was illuminated with photons of different wavelengths, which indistinctly excited DAPI (405 nm laser), Alexa-488 (499 nm laser), Lysotracker™ (576nm laser) and Texas Red (594 nm laser), depending on the experimental design.

#### 4.5.4 Image analysis

To determine the internalization of the different molecules under study in HUVEC, the CellProfiler 4.2.8 release software was used, analyzing the number or the intensity of fluorescent dots in the cytoplasm of the cells.

The quantification of the colocalization analysis was carried out using the colocalization module of the Leica Application Suite X software (Leica Microsystems; Germany) which takes into account the intensities of the fluorophores in each pixel, giving a percentage of colocalization for each image that reflects the degree of colocalization by the overlap of the green and red fluorescent labels. The software calculates the Person colocalization coefficient and gives as a result the percentage of colocalization between the two marks in the whole image analyzed.

### 4.6 Flow cytometry

#### 4.6.1 HUVEC flow cytometry

For determining the cytotoxic effect of histone and blocking endocytosis treatments in HUVEC flow cytometry technique was used.

HUVEC were seeded in 12-well plates, incubating 24 hours at 37 to 37°C and 5% v/v CO2, until they achieved around 80% confluency. Subsequently, the cells were subjected to the appropriate treatments indicated previously, to later analyze their impact on the survival capacity by using an Apoptosis detection kit (Immunostep S.L.; Spain) with Annexin-V labelled with APC (BioLegend, USA) or FITC (Immunostep S.L.; Spain) and propidium iodide (Immunostep S.L.; Spain). To do this, the medium was collected in order to obtain the dead cells which detach from the bottom of the plate, and then the cells which remained attached to the plate were trypsinized and collected with the medium containing dead cells. After that, cells were centrifuged at 2500 rcf for 5 minutes and the supernatant was discarded. Then, cells were resuspended in 100μl of labelling buffer (2-[4-(2-hydroxyethyl) piperazin-1-yl] ethane sulfonic acid/0.1M NaOH (pH 7.4) 1.4M NaCl, CaCl_2_ 25mM) and 5 μl of Annexin-V-APC or Annexin-V-FITC and 5 μl of propidium iodide were added and left to incubate for 15 minutes at room temperature. After this time, the samples were analyzed using a BD FACSVerse™ Cell Analyzer cytometer (BD Biosciences, USA) and FACSuite software. A 561 nm excitation laser was used for propidium iodide, a 488 nm was used for FITC and 633 nm for the APC. The emission wavelengths used were 614 nm for propidium iodide, 525 nm for FITC and 650 nm for APC.

#### 4.6.2 HUVEC Imaging flow cytometry

In order to assess internalization assays within HUVEC, the Amnis^®^ ImageStream^®X^ Mk II Imaging Flow Cytometer (Amnis-Merck Millipore, Germany) was used. HUVEC were seeded in 6-well plates and incubated for 24 hours at 37°C and 5% v/v CO2, until they reached approximately 70% confluency. Then, cells were trypsinized, centrifugated at 2250 rcf for 6 min and resuspended in 2% paraformaldehyde at room temperature (RT) during 15 min. After centrifugation (2500 rcf 6 min), cells were resuspended in PBS with primary antibody H3-Alexa488 at 4°C for 1 h in core histones experiments. Another centrifugation round was permformed, and cell nuclei were stained with 7-AAD viability dye (Beckman Coulter, EE. UU) for 15 min at RT. At least 5000 events were acquired for each condition in each replicate using INSPIRE^®^ software (ImagesStreamX Mark; Amnis-Merck Millipore). Channel 2 (505–560 nm) was used to detect molecules labelled with the Alexa-488 fluorophore, while in core histones experiments channel 5 (642–745 nm) was used to visualize cell nuclei. The laser power used was 65 mW. Magnification 40x. Individual staining tubes were run for each antibody and the compensation matrix provided by IDEAS^®^ software (ImagesStreamX Mark; Amnis-Merck Millipore) was used. The analysis was carried out with IDEAS^®^ software, using the internalization wizard. Additionally, in core histone experiments, the similarity feature was also applied to differentiate extracellular histones from nuclear ones (specifically histone H3).

#### 4.6.3 BVOs flow cytometry

For the flow cytometry analysis of BVOs, first they were exposed to appropriate treatment explained before. Around 20 organoids per condition were pooled for flow cytometry analysis. After the treatments, the organoids corresponding to each condition were collected into a 15mL tube using a cut P1000 tip. As soon as the organoids precipitated to the bottom of the tube, the supernatant was removed and the organoids were washed twice with PBS. 5mL of pre-filtered, pre-warmed enzymatic mix (0.4 mg/mL Liberase TH (Merck, Germany), 3 mg/mL Dispase II (Thermo Scientific, USA) in PBS) was added to the organoids. The organoids were incubated in the enzymatic mix for 25 minutes at 37°C followed by two trituration steps after 10 minutes each. Following, the organoids were filtered through a 70 µm cell strainer and topped up with 45 mL of enzymatic inhibition medium (DMEM + 10% FBS) to stop the enzymatic reactions and recover cells from the strainer. Cells were spun for 10 min 300g 4°C to obtain a pellet. The supernatant was removed and 100 µl of PBS was used to resuspend the cells and transfer them to a 96-V bottom well plate. The plate was centrifuged for 5 min at 450 g and cells were resuspended in 50 µl of diluted Fixable Viability Dye eFluor™ 780 (Invitrogen, USA) per well (1:1000 dilution in PBS). The plate was incubated at 4°C for 30 min and the samples were transferred into FACS tubes for analysis in a LSRFortessa flow cytometer (BD Biosciences, USA). A 633 nm excitation laser was used for APC and a 488 was used for FITC. The emission wavelengths used were 650 nm for APC and 525 for FITC. Data analysis was carried out using FlowJo software (BD Bioscience, USA).

### 4.7 Statistical analysis

A descriptive analysis of the results was carried out using the GraphPad Prism statisticaluio890p program (GraphPad Software; USA), calculating the parameters as mean and standard deviation. The data has been represented as the average of at least three biological experiments or independent replicates. Two-tailed t-Student test were carried out for the comparison of two groups. One-way or two-way ANOVA followed by *post hoc* test (Tukey’s test) were performed for comparisons between groups, considering statistically significant differences from p<0.05. The asterisks that appear in the graphs show the level of significance of the comparisons, representing * when p<0.05, ** when p<0.01, *** when p< 0.001 and **** when p<0.0001.

## Data Availability

The raw data supporting the conclusions of this article will be made available by the authors, without undue reservation.
