# Supplementary material for: Endocytosis and non-canonical autophagy mediate extracellular histones cytotoxicity in vascular models of sepsis
Source: Front Immunol. 2026 Jan 14;16:1650789. doi: 10.3389/fimmu.2025.1650789 (PMC12847238; doi:10.3389/fimmu.2025.1650789)
Supplement: Supplementary file 1 [file Image1.pdf]

SUPLEMENTARY MATERIAL

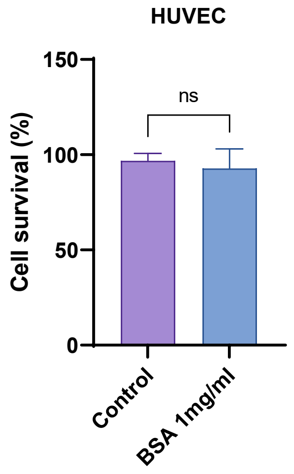

Figure S1.- Analysis of Bovine Serum Albumin (BSA) cytotoxicity on HUVEC. Cells were incubated in the absence (control) or presence of BSA at 1mg/ml for 4 hours. After the indicated incubation time, the cells were harvested and labelled using an Annexin V Kit (, and subsequently analysed by flow cytometry. The mean values are shown as bars, expressed as a percentage ± the standard deviation (n=3). Statistical significance was considered at a p-value < 0.05, T-test, Mann-Whitney statistical test (ns: non-significant).
