# Supplementary material for: Endocytosis and non-canonical autophagy mediate extracellular histones cytotoxicity in vascular models of sepsis
Source: Front Immunol. 2026 Jan 14;16:1650789. doi: 10.3389/fimmu.2025.1650789 (PMC12847238; doi:10.3389/fimmu.2025.1650789)
Supplement: Supplementary file 2 [file Image2.pdf]

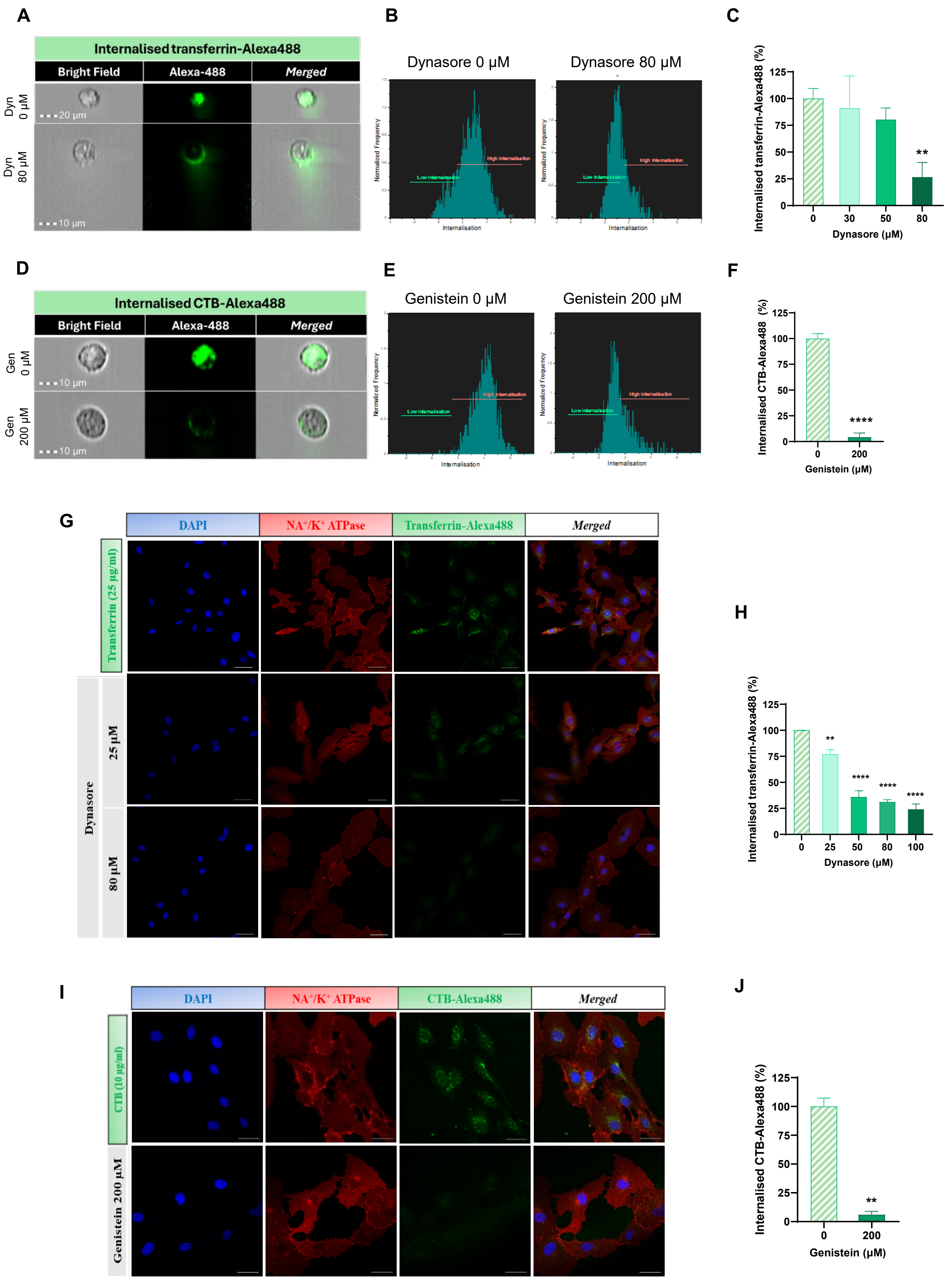

Figure S2.- Optimization of dynasore and genistein experimental conditions in HUVEC cells using transferrin conjugated to Alexa488 and the B subunit of cholera toxin-Alexa-488 as controls (see Figure legend in next page)

Figure S2.- Optimization of dynasore and genistein experimental conditions in HUVEC cells using transferrin conjugated to Alexa488 and the B subunit of cholera toxin-Alexa-488 as controls. A) and D) Representative images of transferrin-Alexa488 (A) or CTB-Alexa488 (D) internalisation in HUVEC cells incubated with different concentrations of dynasore or genistein obtained with the INSPIRE software of the Amnis imaging flow cytometer. Magnification 40X. 10 and 20 µm scale bars. B) and E) Histograms generated using with the internalisation wizard of the INSPIRE software, distinguish between two types of cell populations: in green cells with low internalisation and in red those with higher levels of internalization. C) and F) Quantification of transferrin-Alexa488 (C) or CTB-Alexa488 (F) internalised in HUVEC cells using the internalization algorithm provided by IDEAS software. Mean values are shown as bars, expressed as a percentage ± the standard deviation (n=3). Statistically significant differences were considered when $p<0.05$ (One-way Anova or T-test), in reference to the control (dynasore/genistein 0 µM). G) and I) Confocal microscopy images of HUVEC cells cultured with 25 µg/ml transferrin-Alexa488 (G) or 10 µg/ml CTB-Alexa488(I) under control conditions (no inhibition) and with different concentrations of dynasore or genistein. Blue fluorescence corresponds to cell nuclei by staining with DAPI, red to Na^+/K^+ATPase by binding to a secondary antibody conjugated to Texas Red fluorophore and green to transferrin-Alexa488 or CTB-Alexa488. In addition, a fourth column with the fusion of the three channels used is shown. Magnification 40X (G) and 63X (I); 40 µm scale bars. H) and J) Quantification of transferrin-488 (H) or CTB-488 (J) internalisation in HUVEC cells by measuring the intensity of transferrin-488 (H) or the number of spots (CTB-488) located inside the cell (J) using the Cell Profiler 4.2.8 release software.
