# Supplementary material for: Endocytosis and non-canonical autophagy mediate extracellular histones cytotoxicity in vascular models of sepsis
Source: Front Immunol. 2026 Jan 14;16:1650789. doi: 10.3389/fimmu.2025.1650789 (PMC12847238; doi:10.3389/fimmu.2025.1650789)
Supplement: Supplementary file 3 [file Image3.pdf]

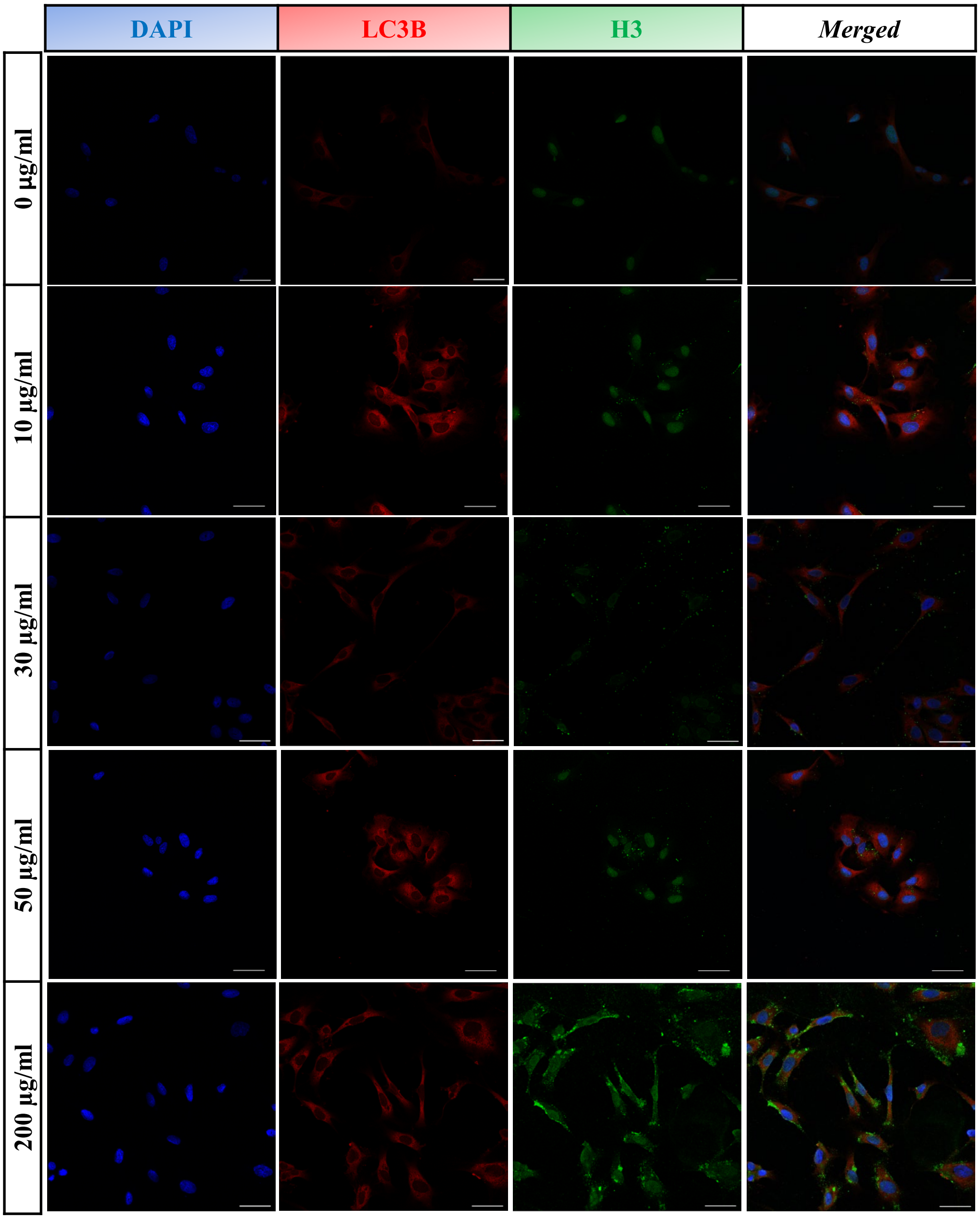


**Figure S3. Colocalization between commercial histone H3 and LC3B.** Representative confocal microscopy images of HUVEC cells treated with commercial histones (mixture of core histones H2AB, H3 and H4) at concentrations between 10 and 200 µg/ml. Each row collects images from the same field of view. Blue fluorescence corresponds to cell nuclei by staining with DAPI, red to LC3B protein by binding to a secondary antibody conjugated to Texas Red fluorophore and green to histone H3 by staining with a secondary antibody conjugated to Alexa488. In addition, a fourth column is shown with the fusion of the three channels used (40 µm scale bars).
